# Inhibitory Effects of Extracellular Vesicles Derived from *Sargassum serratifolium* on Melanogenesis Partially by Suppressing Melanin Transport

**DOI:** 10.3390/md24100344

**Published:** 2026-09-30

**Authors:** Jaeseong Seo, Kyoung Mi Moon, Hyeon Hak Jeong, Su-Yeon Park, Ahreum Kim, Ji Young Hwang, Seonhwa Hwang, Min Hi Park, Won-Kyo Jung, Sang-Hyug Park, Hyeung-Rak Kim, Sung-Han Jo, Minsup Lee, Bonggi Lee

**Affiliations:** 1 Department of Food Science and Nutrition, Pukyong National University, Daeyeon-dong, Nam-gu, Busan 48513, Republic of Korea; syj5528@pukyong.ac.kr (J.S.); omkksm@pknu.ac.kr (K.M.M.); jhh0531@pukyong.ac.kr (H.H.J.); hjy0506@pukyong.ac.kr (J.Y.H.); hrkim@pknu.ac.kr (H.-R.K.); 2 Department of Smart Green Technology Engineering, Pukyong National University, Busan 48513, Republic of Korea; suzan@pukyong.ac.kr (S.-Y.P.); kimar0107@pukyong.ac.kr (A.K.); 3 College of Pharmacy, Kyungsung University, Busan 48434, Republic of Koreaparkmh@ks.ac.kr (M.H.P.); 4 Brain Busan 21 Plus Research Project Group, Kyungsung University, Busan 48434, Republic of Korea; 5 Marine Integrated Biomedical Technology Center, The National Key Research Institutes, Pukyong National University, Busan 48513, Republic of Korea; wkjung@pknu.ac.kr; 6 Major of Biomedical Engineering, Division of Smart Healthcare, College of Information Technology and Convergence and New-Senior Healthcare Innovation Center (BK21 Plus), Busan 48513, Republic of Korea; 7 Department of Industry 4.0 Convergence Bionics Engineering, Pukyong National University, Busan 48513, Republic of Korea; shpark1@pknu.ac.kr (S.-H.P.); josunghan91@gmail.com (S.-H.J.); 8 Major of Biomedical Engineering, Division of Smart Healthcare, College of Information Technology and Convergence, Pukyong National University, Busan 48513, Republic of Korea; 9 Department of Biomedical Engineering, Konyang University, Daejeon 35365, Republic of Korea; 10 Department of Molecular and Cellular Physiology, Louisiana State University Health Shreveport, Shreveport, LA 71103, USA; minsup.lee@lsuhs.edu

**Keywords:** extracellular vesicles, upcycling, skin whitening, melanosome transport

## Abstract

*Sargassum serratifolium *(*S. serratifolium*), listed in the Food Code, is known for its antioxidant and anti-inflammatory properties and is used in cosmetic applications. However, due to limited industrial utilization and overaccumulation in marine environments, *Sargassum* has been designated as a marine pollutant by the UN Environment Agency. To improve resource efficiency and reduce environmental burden, this study developed a dual-extraction strategy to isolate extracellular vesicles (EVs) and extract ethanol-soluble substances from the same *S. serratifolium* biomass. The extracellular vesicles derived from *S. serratifolium* (SEVs) were isolated and characterized based on their particle size, morphology, concentration, and cargo molecules, including mRNA and proteins. Their antioxidant and anti-inflammatory activities were evaluated using cellular assays, while their effects on melanogenesis and melanosome transport were assessed in B16F10 melanoma cells. Transcriptomic analysis by RNA sequencing was further performed to investigate the molecular pathways involved in SEV-mediated regulation of melanosome transport. SEVs isolation did not affect the yield or biological activity of the *S. serratifolium* ethanol-soluble fraction. Based on our previous finding that the ethanol extract of *S. serratifolium *(ESS) suppresses melanin synthesis, the present study focused on investigating the effects and mechanisms of SEVs on melanogenesis and melanosome transport. SEVs were nanoscale vesicles containing mRNA and proteins and showed antioxidant and anti-inflammatory effects in cellular assays. Although SEVs did not reduce melanin production in B16F10 cells, they significantly inhibited melanosome transport. RNA sequencing suggested that SEVs regulated melanosome transport through pathways related to the extracellular matrix, actin filament organization, and calcium ion homeostasis. These findings suggest that SEVs exert anti-melanogenic effects by targeting melanosome transport, providing a complementary mechanism to the previously reported inhibition of melanin synthesis by ESS. These findings demonstrate a sustainable strategy for producing functional EVs and bioactive extracts from a single *S. serratifolium* biomass source and support their potential use in cosmetic or therapeutic applications targeting hyperpigmentation.

## 1. Introduction

Extracellular vesicles (EVs) are nanoscale lipid bilayer-enclosed vesicles secreted by cells [1]. They function as natural carriers of biologically active molecules such as proteins, lipids, and nucleic acids, and play an important role as mediators of cell-to-cell communication [2,3]. Plant-derived EVs (PDVs) share similar properties to mammalian EVs and can effectively interact with mammalian cells to deliver their cargoes for therapeutic effects [4]. Studies have highlighted the efficacy of PDVs in a variety of applications, including anticancer, anti-inflammation, anti-melanogenesis, and osteoporosis prevention [5,6,7,8]. Their biocompatibility, scalability for mass production, and potential as direct therapeutics make PDVs a valuable tool in biomedical applications [9]. Likewise, EVs derived from marine organisms, particularly seaweeds, have demonstrated potential in various biological activities [10]. Recent studies have further highlighted the potential of algae-derived EVs and related nanoscale systems for biomedical applications. Extracellular vesicles isolated from microalgae have been proposed as sustainable and natural nanocarriers of bioactive compounds [11]. Algae-based nanoparticles have also been explored as delivery platforms, particularly for oral drug delivery [12]. More recently, microalgae-derived vesicles have been shown to carry both endogenous and exogenous proteins, further supporting their potential as natural nanocarriers [13]. Seaweed-derived EVs are associated with beneficial properties such as anti-aging effects [14,15], particularly in skin-related applications. Despite these advances, EVs derived from macroalgae, particularly *Sargassum*, remain relatively underexplored.

*Sargassum*, a genus of brown algae, consists of approximately 400 species distributed in marine environments worldwide [16]. Known for its diverse biological activities, including antioxidant, anti-inflammatory, and anti-cancer properties [17,18,19], *Sargassum* has been traditionally consumed as a medicinal food in East Asian countries such as Korea and China [20]. Its multifaceted health benefits and therapeutic potential have been well documented [21]. However, despite these beneficial properties, the potential applications of Sargassum remain underexplored, while its excessive accumulation has become an environmental concern in some regions [22,23]. Large accumulations of *Sargassum* on beaches have negatively affected tourism and marine ecosystems [24]. Additionally, locally accumulated *Sargassum* releases hydrogen sulfide and ammonia, threatening public health and causing economic difficulties due to the cost of removal and disruption of fishing activities [25,26]. Thus, the United Nations Environment Programme has designated *Sargassum* as a marine pollutant due to its excessive accumulation, which negatively impacts marine ecosystems and coastal communities [22]. Therefore, understanding, development, and research of *Sargassum* have the potential to address marine pollution issues and health-related applications. Among the species in this genus, *Sargassum serratifolium* (*S. serratifolium*) has attracted attention for its powerful biological effects [27]. The ethanol extract of *S. serratifolium* (ESS) exhibits potent antioxidant and anti-inflammatory properties and exhibits various physiological activities, including inhibition of muscle atrophy, alleviation of atopic dermatitis, improvement in non-alcoholic fatty liver disease, and inhibition of melanin production via modulation of the CREB/ERK signaling pathway [27,28,29,30,31]. Upcycling *S*. *serratifolium* to extract extracellular vesicles (EVs) not only offers an eco-friendly solution to the environmental challenges posed by marine biomass accumulation but also uncovers novel biological activities distinct from those of its ethanol extract such as antioxidant and anti-inflammatory properties [27,29].

Melanin acts as an important defense mechanism, protecting the skin from ultraviolet (UV) radiation and determining the pigmentation of the skin, hair, and eyes [32,33]. Although essential for protection against UV damage, excessive melanin production can lead to hyperpigmentation disorders such as freckles, blemishes, and age spots [34,35]. These conditions often affect an individual’s appearance and can cause psychological distress [36]. Therefore, understanding and effectively targeting the pathways involved in melanin regulation is important for managing hyperpigmentation-related conditions. Melanin is synthesized within specialized organelles of melanocytes called melanosomes through a process regulated by key enzymes such as tyrosinase, tyrosinase-related protein-1 (TRP-1), and TRP-2 [37]. Tyrosinase plays a central role in the melanogenesis pathway by converting L-tyrosine to L-DOPA, which is then oxidized to form DOPAquinone [38]. Regulation of tyrosinase and related proteins is influenced by α-melanocyte stimulating hormone (α-MSH), which is released in response to UV exposure. α-MSH activates signaling pathways including the cyclic adenosine monophosphate (cAMP) pathway and the extracellular signal-regulated kinase (ERK) mechanism [39], thereby increasing the expression of microphthalmia-related transcription factor (MITF). MITF is a transcription factor for tyrosinase, TRP-1, and TRP-2, which is a key factor in melanogenesis [40]. Existing whitening agents primarily focus on tyrosinase inactivation and inhibition of melanogenesis. However, multiple mechanisms have been shown to contribute to pigmentation regulation. Beyond melanin synthesis, actin-based melanosome transport is essential for pigmentation and transfers melanin from melanocytes to keratinocytes [41]. This process is mediated by a tripartite complex comprising RAB27A, melanophilin (MLPH), and myosin VA (MYO5A), which promotes melanosome transport along actin filaments to the tip of the dendritic spine [42]. In addition, PAR-2-dependent phagocytosis and melanin secretion mediated by the exocyst complex are involved [43,44].

In this study, we isolated and characterized extracellular vesicles derived from *S*. *serratifolium* (SEVs) and investigated their effects on skin-related functions to assess their potential as a bioactive cosmetic ingredient. In parallel, we compared the yield and bioactivity of the ethanol-soluble fraction of *S. serratifolium* obtained with and without prior SEVs isolation to evaluate the feasibility of dual-resource utilization. Because the chemical composition and major bioactive constituents of ethanolic extracts of *S. serratifolium* have been characterized in previous studies [27], untargeted metabolite profiling in the present study was focused on the less-characterized SEV fraction. Furthermore, we examined the combined effects of SEVs and ESS on melanin production and transport, with a particular focus on the role of SEVs in modulating melanosome transport. This integrated approach highlights the potential of *S. serratifolium* as a multifunctional marine resource for pigment regulation and the development of novel strategies for managing hyperpigmentation-related conditions.

## 2. Results

### 2.1. Characterization of SEVs

EVs were isolated from *S. serratifolium* using differential centrifugation and filtration, while ESS was extracted from the residual biomass left after SEVs separation (Figure 1A). The sizes of the isolated SEVs were measured using dynamic light scattering (DLS; Figure 1D). Flow-cytometric analysis using calibrated submicron reference particles provided a complementary estimate of the particle-size distribution. The size distribution ranged from 20 nm to 280 nm, with an average size of 112.9 ± 61.0 nm (Figure 1D). Flow cytometric analysis showed that SEVs were predominantly distributed within the calibrated 100 and 200 nm size ranges. The quantitative results obtained from five independently prepared SEVs samples are presented as the mean ± SD in Figure 2B. TEM images revealed that SEVs were spherical with diameters mostly under 100 nm. Similarly, SEM analysis confirmed their spherical morphology with particle sizes predominantly under 100 nm, consistent with the DLS and flow cytometry results (Figure 1B,C). Since EVs are known to carry biologically active molecules, including genetic material and proteins, we analyzed the RNA and protein cargo of SEVs. After isolating SEVs from 20 g of *S. serratifolium*, RNA was extracted using Trizol, revealing a substantial RNA presence (Figure 1E). Protein content was assessed using a BCA assay, comparing SEVs treated with RIPA buffer and distilled water. The results showed a 31.11% higher protein concentration in SEVs treated with RIPA buffer (Figure 1F), confirming that SEVs were intact and suitable for further experiments.

### 2.2. Untargeted LC-MS/MS Analysis of SEVs

Untargeted LC-MS/MS analysis was performed to profile the metabolic content of SEVs. The detailed extraction procedure is schematically illustrated in Figure 3A. A total of 15 metabolite entries were identified using a local in-house phenolic compound library containing 816 entries, implemented in the UNIFI software (version 3.1.0; Waters). Among these, 13 unique metabolites were identified, with compounds such as Dimethylmatairesinol and Sargachromenol being detected in both positive (POS) and negative (NEG) ionization modes. The distribution of identified compounds across ion modes is shown in the Venn diagram (Figure 3B). Out of the 15 entries, 10 compounds were identified in negative mode, and 5 in positive mode. The observed *m*/*z* values ranged from 111.04 to 610.19, and retention times spanned 2.72 to 22.60 min. Representative base peak intensity (BPI) chromatograms for POS and NEG modes are shown in Figure 3C and D, respectively. Several meroterpenoid compounds—including Sargachromanol C, D, H, J, L, N, Sargachromenol, and sargahydroquinoic acid (SHQA)—were identified, predominantly in the NEG mode. Additionally, flavonoid-related metabolites, such as (-)-Epigallocatechin 3-O-glucuronide, Catechol, and Peonidin 3-O-rutinoside, were identified in POS mode. Details of the identified metabolites—including compound name, molecular formula, observed mass and *m*/*z*, retention time, signal intensity, ion mode, and adduct type—are summarized in Table 1.

### 2.3. SEV’s Antioxidant and Anti-Inflammatory Effects

The antioxidant and anti-inflammatory activities of ethanol-soluble substances derived from *S. serratifolium* have been previously reported [27]. Importantly, the previous study used the same *S. serratifolium* species and the same ethanol extraction procedure used to prepare ESS in the present study. Therefore, we investigated whether SEVs derived from the same species also exhibit antioxidant and anti-inflammatory activities. RAW 264.7 macrophages were used as a well-established cellular model to evaluate LPS-induced inflammatory responses, including NO production, whereas B16F10 cells were used as a melanocyte-related model to evaluate oxidative stress responses. Accordingly, the effects of SEVs on ROS and ONOO^−^ levels were evaluated in LPS-stimulated RAW 264.7 cells and SIN-1-stimulated B16F10 cells, respectively. No significant toxicity was observed in RAW 264.7 macrophages treated with SEVs at up to 50 µg/mL (Figure 4A). SEVs showed antioxidant and anti-inflammatory activities, significantly reducing ROS and ONOO^−^ levels in LPS-stimulated RAW 264.7 macrophages (Figure 4B,C). In B16F10 cells, SEVs did not exhibit significant toxicity up to 150 µg/mL (Figure 4D). In addition, in SIN-1-stimulated B16F10 cells, SEVs significantly reduced ROS and ONOO^−^ levels, indicating antioxidant activity (Figure 4E,F), consistent with the results observed in RAW 264.7 macrophages. These findings suggest that SEVs may possess antioxidant and anti-inflammatory properties, supporting their potential as bioactive cosmetic ingredients.

### 2.4. Effects of SEVs on Melanosome Transport and Melanin Production

We pretreated B16F10 cells with various concentrations of SEVs (10–25 µg/mL) and cultured them with α-MSH for 4 days to evaluate the effects of SEVs on melanogenesis and melanosome transport. To exclude the possibility of delayed cytotoxicity during prolonged SEVs exposure, B16F10 cell viability was additionally evaluated after 4 days of SEVs treatment. No significant cytotoxicity was observed at the tested SEVs concentrations (Figure S2), indicating that prolonged exposure to SEVs did not adversely affect B16F10 cell viability. Compared with the α-MSH group, SEVs treatment increased intracellular melanin content but significantly decreased extracellular melanin levels (Figure 5A,B). However, the reduction in extracellular melanin did not show a clear dose-dependent pattern. Furthermore, microscopic analysis showed that melanin was not distributed to dendritic spines in the SEV-treated group but was mainly retained intracellularly (Figure 5C). These results suggest that the observed changes in melanin content were unlikely to result from delayed cytotoxicity. Together with the intracellular melanin and microscopic observations, the findings suggest that SEVs may affect melanosome transport or release rather than directly inhibiting melanogenesis; however, the extracellular melanin results should be interpreted with caution because a clear dose-dependent response was not observed.

### 2.5. Transcriptomic Changes Associated with SEVs Treatment in α-MSH-Stimulated B16F10 Cells

Based on the observed changes in melanosome distribution and extracellular melanin levels following SEVs treatment, we performed RNA-seq analysis to identify genes whose expression was altered by SEVs treatment. Gene expression profiles were compared between cells treated with α-MSH alone and those treated with α-MSH and SEVs. A total of 495 genes showed significant expression changes (FDR < 0.05 and |log_2_FC| ≥ 0.58). Among these, 197 genes were upregulated, with Srp54b, Slc7a11, Creb5, Lrf6, Sgk1, Unc5b, Pwwp4d, Kcnj13, Prss46, and Popdc2 showing increases in expression. In contrast, 298 genes were downregulated, including Crabp2, Entpd4b, Slc16a3, Mal, Gsta4, Adam12, Ednra, Ptgir, Fbxw15, Col19a1, and Wfdc6a (Figure 6A). To further investigate the biological functions associated with SEVs treatment, Gene ontology analysis was performed on the differentially expressed genes (DEGs). GO biological process (BP) analysis of the downregulated genes revealed enrichment of terms related to extracellular matrix organization, calcium homeostasis, and actin filament-associated processes. Based on their potential relevance to cellular morphology and intracellular transport, eight GO BP terms were selected for further analysis: extracellular matrix organization (GO:0030198), intracellular calcium ion homeostasis (GO:0006874), calcium ion homeostasis (GO:0055074), actin filament polymerization (GO:0030041), collagen-activation signaling pathway (GO:0038065), actin polymerization or depolymerization (GO:0008154), regulation of actin filament-based processes (GO:0032970), and actin filament organization (GO:0007015) (Figure 6B,C). These results indicate that SEVs treatment was associated with changes in broader biological processes involving extracellular matrix organization, calcium homeostasis, and actin filament dynamics. These pathways may indirectly influence melanocyte morphology and intracellular transport. However, the core melanosome transport genes RAB27A, MYO5A, and MLPH were not identified as differentially expressed genes. Therefore, the transcriptomic findings do not directly demonstrate that SEVs inhibit the established melanosome transport machinery.

### 2.6. GO Network Analysis of Down-Regulated Genes Induced by SEVs Treatment in α-MSH-Stimulated B16F10 Cells

In the gene expression pattern of α-MSH-stimulated B16F10 cells, we performed an extensive network analysis to identify additional insights and associations in the functional characteristics of the genes in eight pathways that may be associated with the inhibition of melanosome transport by treatment with SEVs.

In the “Regulation of actin filament-based process” category of GO analysis, significant fold changes were observed in genes such as Epha5, Serpinf2, and Sptb. In the “Intracellular calcium ion homeostasis” category, a notable fold change was observed in the Ednra gene. Similarly, in the “Extracellular matrix organization” category, high fold changes were detected in genes such as Col11a1, Col19a1, Mmp17, Olfml2 b, and Serpinf2. The “Calcium ion homeostasis” category also showed a significant fold change in the Ednra gene. Furthermore, in the “Actin polymerization or depolymerization” and “Actin filament polymerization” categories, high fold changes were observed in Sptb. Lastly, in the “Actin filament organization” category, prominent fold changes were noted in genes such as Serpinf2, Shroom3, and Sptb (Figure 7A,B). These results suggest that SEVs treatment may be associated with changes in extracellular matrix organization, actin filament-associated processes, and calcium ion homeostasis. However, the potential functional relationships between these transcriptional changes and melanosome transport require further investigation.

### 2.7. RT-qPCR Assessment of Selected Candidate Genes

To assess the expression patterns observed in the exploratory RNA-seq analysis, six candidate DEGs—Kank4, Ednra, Pth1r, Prex1, Serpinf2, and Olfml2b—were selected for RT-qPCR analysis based on their expression changes and potential relevance to broader cytoskeletal or cellular signaling processes. Kank4 and Pth1r were included as exploratory candidate DEGs rather than as established regulators of melanosome transport.

Significant downregulation was observed in Kank4, Ednra, and Pth1r, whereas Prex1 showed a decreasing trend without reaching statistical significance (*p* = 0.0620) (Figure 8A–D). No significant differences were observed in Serpinf2 or Olfml2b (Figure 8E,F). Thus, the RT-qPCR results were partially consistent with the expression patterns observed in the exploratory RNA-seq analysis.

### 2.8. The Dual Utilization of SEVs and ESS from S. serratifolium

We evaluated the yield and anti-inflammatory activity of ESS extracted with and without prior SEVs isolation to assess the feasibility of dual utilization of SEVs and ESS from *S. serratifolium*. We obtained 1.12 g of ESS from 8.43 g of dry *S. serratifolium* biomass without SEVs separation, yielding 13.3%. In contrast, 1.98 g of ESS was obtained from 12.16 g of dry *S. serratifolium* biomass following SEVs separation, yielding 16.3% (Table 2). Because different amounts of dry biomass were used for the two extraction conditions, these percentage yields should not be interpreted as a direct quantitative comparison of extraction efficiency. Nevertheless, ESS was recovered from the residual biomass following SEVs isolation, demonstrating the feasibility of sequential recovery of SEVs and ESS from the same biomass.

RAW 264.7 macrophages were selected as a well-established model of LPS-induced inflammatory responses to evaluate whether prior SEVs isolation affected the anti-inflammatory activity of ESS. Accordingly, the anti-inflammatory activity of ESS was evaluated by measuring nitrite accumulation in the culture medium of LPS-stimulated RAW 264.7 macrophages using the Griess assay. ESS prepared from *S. serratifolium* without prior SEVs isolation significantly reduced LPS-induced nitrite accumulation at concentrations of 1–5 μg/mL, whereas ESS prepared after SEVs isolation significantly reduced nitrite accumulation at concentrations of 0.5–5 μg/mL (Figure 9A). Importantly, no significant difference was observed between the two ESS preparations at the concentrations (1–5 μg/mL) at which significant inhibition was observed in both samples. These results suggest that prior SEVs isolation did not adversely affect the anti-inflammatory activity of ESS under the tested conditions. Together with the recovery of ESS from the residual biomass following SEVs isolation, these findings support the feasibility of sequential utilization of SEVs and ESS from *S. serratifolium*.

Previous work has reported the hypopigmenting activity of ethanolic extracts from S. serratifolium and identified sargaquinoic acid (SQA) as an active component. SQA has been shown to suppress melanin production through cAMP/CREB- and ERK1/2-mediated downregulation of MITF in α-MSH-stimulated B16F10 cells [31]. To investigate their potential complementary effects, we evaluated the combined effects of ESS and SEVs on melanogenesis and melanosome transport. ESS significantly reduced intracellular melanin production compared with the α-MSH group but did not significantly affect extracellular melanin levels. On the other hand, SEVs did not significantly reduce intracellular melanin content but were associated with a lower extracellular melanin signal under the tested conditions. Interestingly, combined treatment with ESS and SEVs reduced intracellular melanin content and the measured extracellular melanin signal (Figure 9B,C).

## 3. Discussion

### 3.1. Interpretation and Practical Significance of SEVs Characterization

The differences among the particle-characterization techniques used in this study should be considered when interpreting SEVs size. DLS measures the hydrodynamic diameter of particles in suspension and may yield relatively large size values because of the hydration layer and sensitivity to particle aggregation. In contrast, TEM and SEM provide direct information on particle morphology and dimensions in dried samples, although dehydration and sample-preparation procedures may affect the observed particle size. Flow cytometry was used as a complementary method to estimate particle populations within calibrated size ranges and particle concentration, rather than as the primary method for particle-size determination. From a practical perspective, particle concentration provides a useful parameter for dose standardization, comparison among SEVs preparations, and assessment of batch-to-batch consistency. When reported together with the initial biomass and final preparation volume, it may also assist in evaluating isolation yield and optimizing the production process.

### 3.2. Dual-Use Potential of SEVs and ESS Derived from S. serratifolium

This study highlights the dual-use potential of SEVs and ESS derived from *S. serratifolium* and their complementary roles in pigmentation control. Importantly, SEVs isolation did not compromise the yield or bioactivity of ESS, indicating that both SEVs and ESS can be recovered and utilized from the same *Sargassum* biomass. This dual-resource utilization approach may enhance the value of *Sargassum* biomass and support its sustainable utilization, particularly in regions where *Sargassum* overaccumulation has become an environmental concern.

### 3.3. Distinct and Complementary Mechanisms of SEVs and ESS in Pigmentation Control

Our findings reveal that SEVs and ESS act through distinct yet complementary mechanisms. ESS primarily attenuates melanogenesis via its major bioactive meroterpenoids, SHQA and SQA, which inhibit tyrosinase activity and suppress MITF and its downstream targets through the cAMP/CREB and ERK1/2 pathways [27,31,45]. In contrast, SEVs appear to exert their effects primarily by modulating melanosome transport rather than directly inhibiting melanin synthesis. Although SHQA was detected in both ESS and SEVs, the two preparations showed distinct effects on pigmentation. The identification of SHQA by untargeted LC–MS/MS does not demonstrate that it is present at an equivalent or biologically effective concentration in the two preparations because absolute quantification was not performed. Moreover, ESS contains SQA, SHQA, SCM, and other low-molecular-weight constituents that may contribute individually or collectively to the inhibition of tyrosinase activity and melanin synthesis. In SEVs, SHQA may be encapsulated within the vesicles or associated with their membranes, potentially altering its release, accessibility, and intracellular availability compared with the freely extracted constituents in ESS. However, the localization and bioavailability of SHQA within SEVs were not directly investigated. Therefore, the detection of SHQA in SEVs does not necessarily result in the same direct antimelanogenic activity observed with ESS. The present findings suggest that ESS primarily suppresses melanin synthesis, whereas SEVs predominantly modulate melanosome transport. Further quantitative analyses and experiments using isolated SHQA and SQA are required to establish the specific contribution of these compounds.

Transcriptomic and GO enrichment analyses indicated that SEVs treatment was associated with broader changes in ECM organization, actin filament-associated processes, and calcium ion homeostasis. These changes may indirectly influence melanocyte morphology and intracellular transport; however, they do not establish direct disruption of the canonical melanosome transport machinery [46,47,48,49]. This distinct mechanism suggests that SEVs may complement conventional melanogenesis-targeting agents, including ESS.

### 3.4. SEVs-Derived Metabolites Associated with Calcium Signaling and Melanosome Transport

Untargeted LC-MS/MS analysis further provided insight into the potential molecular basis of the activity of SEVs, identifying 13 bioactive metabolites, including (-)-epigallocatechin 3-O-glucuronide, catechol, dimethylmatairesinol, peonidin 3-O-rutinoside, multiple sargachromanols, sargachromenol, SHQA, and vanillic acid 4-sulfate. Among these, SHQA has been shown to suppress melanogenesis in B16F10 cells [45] and to decrease intracellular Ca^2+^ levels in PMACI-stimulated KU812F cells [50]. These findings are consistent with the GO enrichment of downregulated genes associated with intracellular calcium ion homeostasis (GO:0006874) and calcium ion homeostasis (GO:0055074), both of which are involved in melanin synthesis and melanosome transport [51,52,53]. Collectively, these results support the possibility that SHQA may contribute to the regulation of melanosome transport in α-MSH-stimulated melanocytes by modulating calcium signaling; however, this proposed mechanism requires direct validation in B16F10 cells.

Additional SEVs-derived metabolites may also contribute to the observed effects on melanosome transport. Although vanillic acid 4-sulfate is generally considered less bioactive due to sulfation, its precursor has been reported to restore calcium homeostasis by modulating ER–mitochondrial calcium crosstalk [54]. Similarly, (-)-epigallocatechin 3-O-glucuronide, a conjugated form of EGCG, may partially retain calcium-regulating activity despite glucuronidation [55]. Together with SHQA, these metabolites may potentially contribute to calcium-dependent pathways associated with melanosome transport. In line with this, SEVs treatment downregulated pathways associated with ECM remodeling and actin filament dynamics, which are fundamental for dendrite elongation and melanosome trafficking. These findings suggest a possible association between SEVs treatment and changes in calcium signaling, ECM organization, and cytoskeletal dynamics; however, their direct involvement in melanosome transport requires further functional validation.

### 3.5. Exploratory Transcriptomic Findings Associated with Melanosome Transport

The exploratory transcriptomic analysis identified changes in broader pathways related to ECM organization, actin filament-associated processes, and calcium homeostasis. These pathways may indirectly influence melanocyte morphology and intracellular transport; however, the core melanosome transport genes RAB27A, MYO5A, and MLPH were not identified as differentially expressed genes. RT-qPCR analysis also provided only partial support for the RNA-seq findings. Kank4, Ednra, and Pth1r were significantly downregulated, whereas Prex1 showed a non-significant decreasing trend (*p* = 0.062), and Serpinf2 and Olfml2b showed no significant differences. Therefore, these transcriptomic findings should be considered exploratory rather than direct evidence that SEVs regulate the established melanosome transport machinery.

This process depends on a tripartite protein complex consisting of RAB27A, MYO5A, and MLPH, which drives melanosome movement along actin filaments toward dendrite tips for transfer to keratinocytes [42]. By modulating ECM integrity, actin filament stability, and calcium signaling—a key regulator of cytoskeletal dynamics [46,47,48,49]—SEVs may interfere with this transport system, thereby preventing efficient melanosome transfer. However, the extracellular melanin results should be interpreted with caution because the reduction in extracellular melanin did not exhibit a clear dose-dependent pattern. Moreover, extracellular melanin was quantified directly from the culture supernatant by absorbance measurement, and a separate cell-free SEVs blank was not included. Therefore, potential interference from the intrinsic color or optical properties of SEVs cannot be completely excluded. Although phenol red-containing DMEM was used for all groups under identical conditions, its potential contribution to the absorbance measurement also cannot be completely excluded. This limitation specifically concerns the extracellular melanin assay and does not apply to the intracellular melanin measurement, in which the culture medium was removed before absorbance measurement. Therefore, the extracellular melanin data should be considered together with the intracellular melanin and microscopic observations when interpreting the potential effects of SEVs on melanosome transport. Taken together, the present findings suggest that SEVs may affect cellular processes associated with melanosome transport rather than directly inhibiting melanin synthesis; however, further functional studies are required to establish the underlying mechanism.

### 3.6. Sustainability and Potential Applications of SEVs and ESS

Beyond these mechanistic insights, SEVs derived from *S. serratifolium* present a compelling opportunity to advance both environmental sustainability and functional innovation. SEVs isolation does not reduce the yield or efficacy of ESS, supporting the dual utilization of SEVs and ESS from a single biomass source. This dual-use strategy maximizes resource efficiency and provides an innovative model for sustainable marine resource management aligned with global efforts to address climate change and marine pollution. Moreover, the complementary bioactivities of SEVs and ESS—encompassing pigmentation control, antioxidant capacity, and anti-inflammatory effects—highlight their potential as multifunctional ingredients for cosmetic and pharmaceutical applications. By transforming *S. serratifolium* from an ecological burden into a high-value bioactive resource, this integrated approach unlocks new possibilities in marine biotechnology and paves the way for next-generation skin health solutions.

### 3.7. Potential Relevance of SEV-Mediated Melanosome Transport Regulation to Human Skin

In human skin, epidermal pigmentation depends not only on melanin synthesis but also on melanosome transport within melanocytes and the subsequent transfer of melanosomes to neighboring keratinocytes. A single melanocyte interacts with approximately 36 surrounding keratinocytes, and melanosome transport toward melanocyte dendrite tips is mediated by actin-dependent machinery involving the RAB27A–MYO5A–MLPH complex. Therefore, modulation of melanosome transport may provide a complementary strategy for pigmentation control.

In the present study, SEVs altered melanocyte dendritic morphology and the expression of genes associated with calcium homeostasis, extracellular matrix organization, and actin filament dynamics, suggesting their potential involvement in melanosome transport. However, these findings were obtained using B16F10 melanoma cells, and their relevance to human skin has not yet been directly established. Human melanocytes, melanosome transfer to keratinocytes, skin penetration, irritation, and clinical efficacy were not evaluated in the present study. Therefore, the potential use of SEVs in topical or cosmeceutical formulations should be regarded as a future application rather than an established effect. Further studies using human melanocytes, melanocyte–keratinocyte co-cultures, three-dimensional human skin models, and in vivo systems are required to determine the efficacy, safety, and skin-delivery properties of SEVs.

### 3.8. Conclusions

This study demonstrated that SEVs and ESS derived from *S*. *serratifolium* possess distinct but complementary activities in pigmentation control. SEVs primarily affected melanosome transport, whereas ESS exerted inhibitory effects on melanin synthesis. Targeting melanosome transport may provide a complementary approach to pigmentation control; however, further studies using human skin-relevant models are required to establish its efficacy and safety.

Importantly, SEVs isolation did not adversely affect the yield or anti-inflammatory activity of ESS, supporting the feasibility of their dual utilization from a single *Sargassum* biomass source. Transcriptomic and metabolomic analyses further suggested that SEV-mediated regulation of melanosome transport may involve calcium signaling, extracellular matrix organization, and actin filament dynamics. These findings provide new insights into the biological activities of *S. serratifolium*-derived SEVs and support their potential, together with ESS, as multifunctional ingredients for cosmetic applications. Further studies, including mechanistic validation and in vivo investigations, are warranted to clarify their biological mechanisms and application potential.

## 4. Materials and Methods

### 4.1. Materials

2′,7′-Dichlorodihydrofluorescein diacetate (DCF-DA), dihydrorhodamine 123 (DHR 123), diethylenetriaminepentaacetic acid (DTPA), Morpholinosydnonimine hydrochloride (SIN-1 hydrochloride), α-melanocyte stimulating hormone (α-MSH), lipopolysaccharide (LPS, *E. coli* O111:B4), and dimethyl sulfoxide (DMSO) were purchased from Sigma-Aldrich (St. Louis, MO, USA).

### 4.2. Isolation and Protein Quantification of Extracellular Vesicles from S. serratifolium

*S. serratifolium* used in this study was provided by Professor Hyeung-Rak Kim of Pukyong National University and originated from the same collection previously reported by Gwon et al. [56]. The taxonomic identity of the specimen was confirmed by C.G. Choi, an algal taxonomist in the Department of Ecological Engineering at Pukyong National University (Busan, Republic of Korea). A voucher specimen was deposited in the Marine Brown Algae Resources Bank, Republic of Korea, under voucher number TC18835 [56]. Extracellular vesicle (EV) isolation was performed using a slightly modified previously published method [5]. The collected *S. serratifolium* was thoroughly washed, and mixed with phosphate-buffered saline (PBS), and the tissue was ground using a food blender. The sample was centrifuged at 5000× *g* for 1 h and then at 12,000× *g* for 2 h to remove large tissues and insoluble materials. The resulting supernatant was filtered through a 0.22 μm membrane to remove residual particles. The supernatant was then concentrated using an Amicon Ultra-4 PL 100 K centrifugal filter (Merck Millipore, Darmstadt, Germany) by centrifugation at 5000× *g* for 30 min. All centrifugation procedures were performed at 4 °C. Isolated EVs were suspended in PBS, quantified using the Pierce™ BCA Protein Assay Kit (Thermo Fisher Scientific, Waltham, MA, USA), and stored at −70 °C until use.

Protein concentration was determined using a BCA protein assay based on a standard curve. For protein quantification, 10 μL of each sample was used for the assay.

### 4.3. SEVs Morphology Analysis

TEM images of SEVs were acquired using a JEM-F200 TEM (JEOL, Tokyo, Japan), and SEM images were obtained using a ZEISS Crossbeam 550 (Carl Zeiss, Oberkochen, Germany). SEVs suspensions were loaded onto carbon-coated grids (TED PELLA, Redding, CA, USA) and dried in a fume hood. The samples were fixed with 4% paraformaldehyde (PFA) for 30 min and washed twice with PBS. They were then dehydrated in an ascending ethanol series (50%, 70%, 90%, 95%, 100%) and dried in a fume hood.

### 4.4. Size Characterization of SEVs

The size distribution of SEVs was determined by dynamic light scattering (DLS) using a Litesizer 500 (Anton Paar, Graz, Austria). DLS measurements were performed by diluting SEVs suspensions 1:100 with distilled water, transferring them to cuvettes, and measuring at 25 °C. Results are presented as size distribution graphs based on triplicate measurements. Additionally, the size distribution and particle concentration of SEVs were analyzed using a CytoFLEX flow cytometry with CytExpert software, version 2.4 (Beckman Coulter, Brea, CA, USA), with PBS used as a negative control. Calibration was performed using a flow cytometry submicron particle size reference kit to ensure accurate measurement of vesicle size within the nanometer range.

### 4.5. Extraction of ESS from S. serratifolium

*S. serratifolium* with and without SEV isolation were air-dried and ground. Each ground sample was mixed with 70% ethanol (1:15, *w*/*v*) and extracted at 76 °C for 24 h. The extracts were filtered under reduced pressure using filter paper (Whatman, Kent, UK). The extraction residues were re-extracted with 70% ethanol (1:10, *w*/*v*) at 76 °C for an additional 24 h. The first and second extracts were concentrated using a rotary vacuum evaporator and lyophilized in a freeze dryer (FDU-2100, EYELA, Tokyo, Japan) at −80 °C for 72 h. The resulting powder was stored at −20 °C until further use.

### 4.6. Extraction of SEVs Contents for LC-MS/MS Analysis

The extraction of metabolites from SEVs derived from *S. serratifolium* was performed with reference to previously reported plant-based extracellular vesicle studies, with slight modifications to optimize recovery. Starting with the SEVs suspension in PBS (prepared as described in Section 4.2), SEVs were pelleted by centrifugation at 20,000× *g* for 30 min at 4 °C (up to 2 h when needed), and the supernatant (PBS) was discarded. The SEVs pellet was resuspended in 1 mL of extraction solvent consisting of methanol and ultrapure water (4:1, *v*/*v*). The suspension was sonicated at 40 kHz in a 5 °C water bath for 30 min to promote metabolite release. Following sonication, the samples were centrifuged at 13,000× *g* for 15 min at 4 °C. The resulting clear supernatant was carefully transferred into LC-MS sample vials and used for untargeted metabolomic profiling. These steps are visually represented in Figure 3A.

### 4.7. Untargeted Metabolomic Analysis of SEVs Using LC-MS/MS

Untargeted LC-MS/MS analysis was performed using a tandem quadrupole mass spectrometer (XEVO TQ-S micro, Waters Corporation, Milford, MA, USA) coupled with an ultra-performance liquid chromatography (UPLC) system. Metabolites were separated on a BEH C18 column (Waters Corporation, Milford, MA, USA) at a flow rate of 0.3 mL/min. The mobile phases consisted of (A) water with 0.1% formic acid and (B) acetonitrile with 0.1% formic acid. A solvent gradient was applied according to the program shown in Supplementary Table S1. Mass spectrometric detection was performed in both positive and negative ion modes over an m/z range of 50–1200. Instrumental parameters and source settings were optimized based on the manufacturer’s recommendations. Compound identification was performed using the UNIFI software (Waters), employing a custom in-house library containing 816 phenolic compounds. Matching was based on retention time, exact mass, isotope pattern, and adductions under both positive and negative ionization modes. This analysis was intended for qualitative metabolite profiling; absolute concentrations were not determined.

### 4.8. Cell Cultures

Cell culture procedures were performed according to previously described methods [57]. B16F10 mouse melanoma cells were obtained from the American Type Culture Collection (ATCC, Manassas, VA, USA), and the RAW 264.7 cells were obtained from the Korean Cell Line Bank (Seoul, Korea). Both B16F10 and RAW 264.7 cells were cultured in Dulbecco’s Modified Eagle’s Medium (DMEM) containing 10% heat-inactivated fetal bovine serum (FBS) and 1% penicillin–streptomycin (P/S) at 37 °C in 5% CO_2_.

### 4.9. Cell Viability Assay

Cell viability was assessed based on a previously reported method [58]. B16F10 cells (1 × 10^4^ cells/well) and RAW 264.7 macrophages (4 × 10^4^ cells/well) were seeded in 96-well plates. After 24 h, B16F10 cells and RAW 264.7 macrophages were treated with SEVs at 5–200 μg/mL concentrations. After 24 h of incubation, 5 μL of MTS reagent (CellTiter96 AQueous One Solution Cell Proliferation Assay Kit, Promega, Madison, WI, USA) was added to each well. After 1 h, absorbance was measured at 490 nm using a microplate reader. To evaluate potential delayed cytotoxicity following prolonged exposure to SEVs, B16F10 cell viability was additionally assessed after 4 days of treatment with SEVs at 5, 10, 20, 50,100 and 150 μg/mL. Cell viability was determined using the same MTS assay described above.

### 4.10. Measurement of Nitrite Using the Griess Assay

The quantification of nitrite as an indirect indicator of NO production was performed as previously described [30]. RAW 264.7 macrophages were seeded in a 96-well plate at a density of 4 × 10^4^ cells/well. After 24 h, the cells were treated with ESS extracted from *S. serratifolium*, processed with or without the SEVs isolation process, at various concentrations (0.5–5 μg/mL). One hour after ESS treatment, LPS (500 ng/mL) was added to each well. After 24 h, 100 μL of the supernatant was collected and diluted 1:1 with Griess reagent, consisting of 8.5% phosphoric acid, 1% sulfanilamide, and 0.1% naphthylethylenediamine dihydrochloride. The mixture was incubated for 30 min, and the absorbance was measured at 490 nm using a microplate reader.

### 4.11. ROS and ONOO^−^ Scavenging Activity

ROS and ONOO^−^ scavenging activities were measured using fluorescent probes DCFDA and DHR 123, respectively, according to a previously detailed method [59]. B16F10 cells and RAW 264.7 macrophages were seeded in black 96-well plates at a density of (1 × 10^4^ cells/well). After 24 h, the cells were pretreated with various concentrations of SEVs (1–50 μg/mL) and subsequently exposed to 1 mM SIN-1 for B16F10 cells or 500 ng/mL LPS for RAW 264.7 macrophages for an additional 24 h. After the incubation period, the cell culture medium was removed. For ROS measurement, each well was treated with 150 μL of 0.125 mM DCF-DA diluted in PBS. For ONOO^−^ measurements, each well was filled with 180 μL of reaction buffer containing rhodamine solution (50 mM sodium phosphate buffer, 90 mM sodium chloride), 50 mM DTPA, and 5 μM DHR 123. Fluorescence changes were monitored using a Cell Imaging Multi-Mode Reader (Cytation 5; BioTek, Winooski, VT, USA) at excitation and emission wavelengths of 485 nm and 535 nm, respectively. Measurements were recorded every 5 min for 30 min.

### 4.12. Melanin Contents in B16F10 Melanoma Cells

Melanin contents in B16F10 melanoma cells were determined in both intracellular and extracellular fractions, following a previously described method [57]. B16F10 cells were seeded in Petri dishes (60 × 15 mm) at a density of 5 × 10^4^ cells/dish and cultured for 24 h. The cells were pretreated with ESS (10 μg/mL), SEVs (17.5 μg/mL), ESS + SEVs, or Arbutin (500 μM). One hour later, 1 μM α-MSH was added, and the cells were cultured for an additional 4 days. For intracellular melanin measurement, the culture medium was removed, and the cells were washed and collected by centrifugation. The resulting cell pellets were lysed in 1 N NaOH solution at 60 °C for 1 h, and the dissolved melanin was measured at 409 nm using a microplate reader. For extracellular melanin measurement, culture supernatants were directly collected, and the absorbance was measured at 409 nm. The untreated control consisted of culture supernatant collected from B16F10 cells maintained in the same phenol red-containing DMEM used for all experimental groups. A separate cell-free medium blank was not included in the assay. All melanin contents were normalized to the protein content determined by a BCA assay.

### 4.13. SEVs Genetic Material Analysis and Real-Time PCR

To determine the RNA concentration of SEVs, RNA was extracted using the RiboEX reagent (GeneAll, Seoul, Republic of Korea), eluted in a final volume of 50 μL, and subsequently measured with a Nabi UV/Vis Nano Spectrophotometer (MicroDigital, Seongnam, Korea). RNA was extracted from cells using the RiboEX reagent. cDNA synthesis was carried out with the SmartGene Compact cDNA Synthesis Kit (SMART GENE, Daejeon, Republic of Korea). Quantitative real-time PCR (qPCR) was then performed using the TOPreal SYBR Green qPCR PreMIX (Enzynomics, Daejeon, Republic of Korea) on the QuantStudio 1 Real-Time PCR system (Applied Biosystems, Foster City, CA, USA). The GAPDH gene was used as the reference for normalization, and gene expression levels were analyzed using the 2^−ΔΔCT^ method.

### 4.14. RNA Purification and Sequencing

RNA preparations with RIN ≥ 7.0 were used to prepare libraries using the Illumina TruSeq Stranded mRNA Sample Prep Kit (Illumina, Inc., San Diego, CA, USA, #RS-122-2101). The libraries were prepared following the qPCR Quantification Protocol Guide (KAPA Biosystems, Wilmington, MA, USA; catalog no. KK4854). Indexed libraries were then submitted to Illumina NovaSeqX (Illumina, Inc., San Diego, CA, USA) and paired-end (2 × 100 bp) sequenced. They were then mapped to the reference genome using HISAT2 (version 2.2.1) [30,60]. Read counts were generated using the featureCounts program (version subread-2.0.8) [61].

### 4.15. Analysis of Differentially Expressed Genes

Differential expression analysis was performed following a method adapted from previous studies [30]. Briefly, TMM normalization was conducted using the edgeR package (version 4.0.16). The normalization employed the no replicate method outlined in the edgeR: Differential Analysis of Sequence Read Count Data User’s Guide, with a square root dispersion value of 0.1. Genes exhibiting a log2 fold change (FC) ≥ 0.58, ≤ −0.58, and an FDR < 0.05 were identified as differentially expressed and subsequently subjected to gene ontology enrichment analysis.

### 4.16. Statistical Analysis

The experimental data were obtained from at least three independent experiments and are expressed as the mean ± standard deviation (SD). Statistical analyses were performed using a paired t-test for comparisons between two groups and one-way analysis of variance (ANOVA) followed by Tukey’s multiple-comparison test for comparisons among multiple groups. All statistical analyses were performed using GraphPad Prism 5.0 (GraphPad Software, La Jolla, CA, USA). A *p*-value < 0.05 was considered statistically significant.

## Figures and Tables

**Figure 1:**
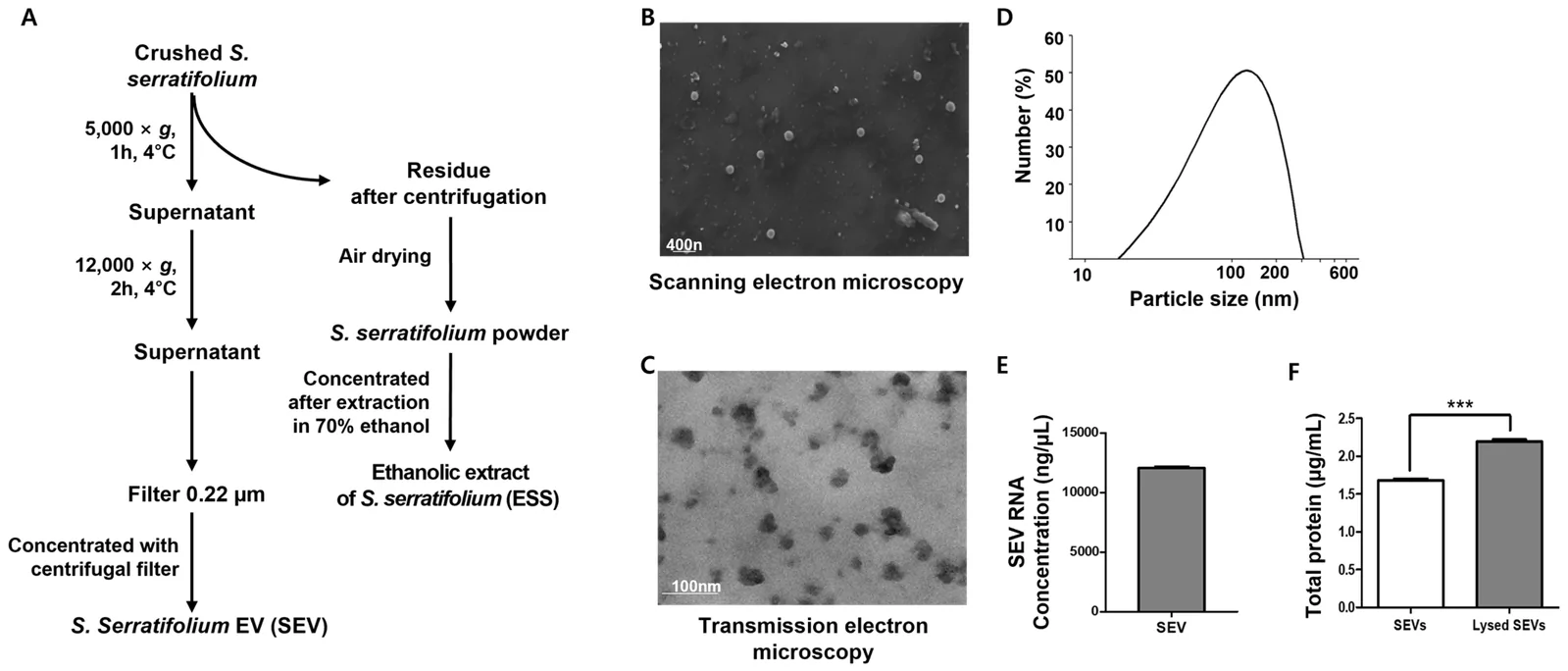
Characterization of SEVs. (**A**) Schematic diagram of SEVs isolation and ethanol extraction protocol from *S. serratifolium*. (**B**) Morphology of SEVs identified using scanning electron microscopy (SEM). Scale bar = 400 nm. (**C**) Morphology of SEVs identified using transmission electron microscopy (TEM). Scale bar = 100 nm. (**D**) The size distribution of SEVs was measured using dynamic light scattering (DLS). (**E**) Concentration of RNA extracted from SEVs isolated from 20 g of *S. serratifolium*, as determined by absorbance measurement. (**F**) Total protein concentrations of water-treated and RIPA-lysed SEVs were measured using a BCA assay. Data are presented as mean ± SD, and statistical significance was determined using a paired *t*-test. *** *p* < 0.001 compared with the distilled water group.

**Figure 2:**
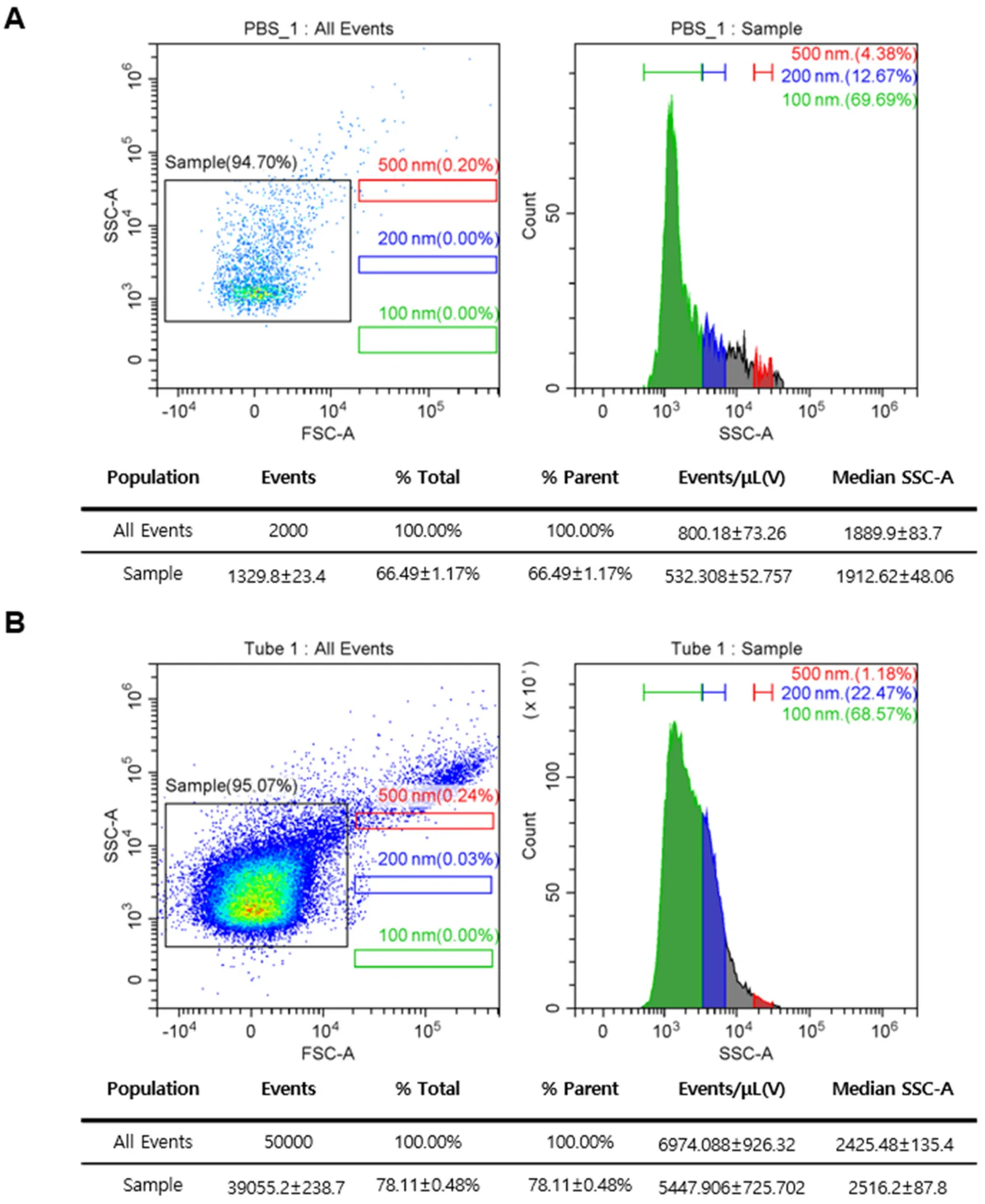
Flow cytometry-based analysis of SEV size distribution and particle concentration. (**A**) Flow cytometry profile of PBS used as a negative control. (**B**) The size distribution and particle concentration of SEVs isolated from *S. serratifolium*. Particle size calibration was performed using a Flow Cytometry Submicron Particle Size Reference Kit. Quantitative data are presented as the mean ± SD of five independently prepared samples of SEVs (*n* = 5).

**Figure 3:**
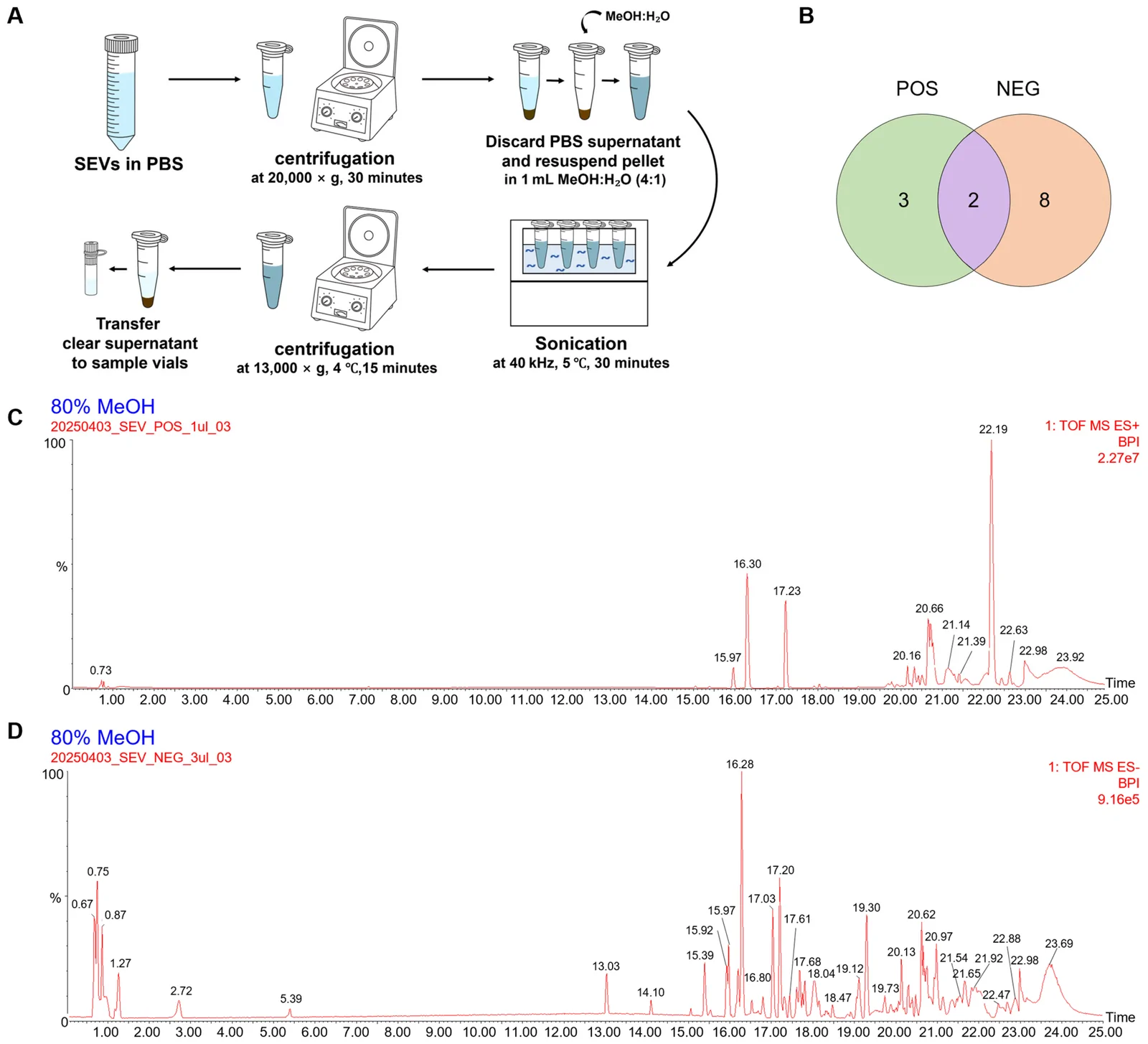
LC-MS/MS-based metabolite analysis of SEVs from *S. serratifolium.* (**A**) Schematic workflow illustrating the extraction process of small extracellular vesicle (SEVs) contents from *S. serratifolium* for LC-MS/MS analysis. (B) Venn diagram showing the distribution of identified metabolites in positive (POS) and negative (NEG) ion modes. Green represents metabolites detected only in POS mode, orange represents metabolites detected only in NEG mode, and purple represents metabolites detected in both modes. The identification was performed using a custom in-house library containing 816 phenolic compounds. (**C**) Base peak intensity (BPI) chromatogram of SEV-derived metabolites acquired in positive ion mode. (**D**) BPI chromatogram of SEV-derived metabolites acquired in negative ion mode.

**Figure 4:**
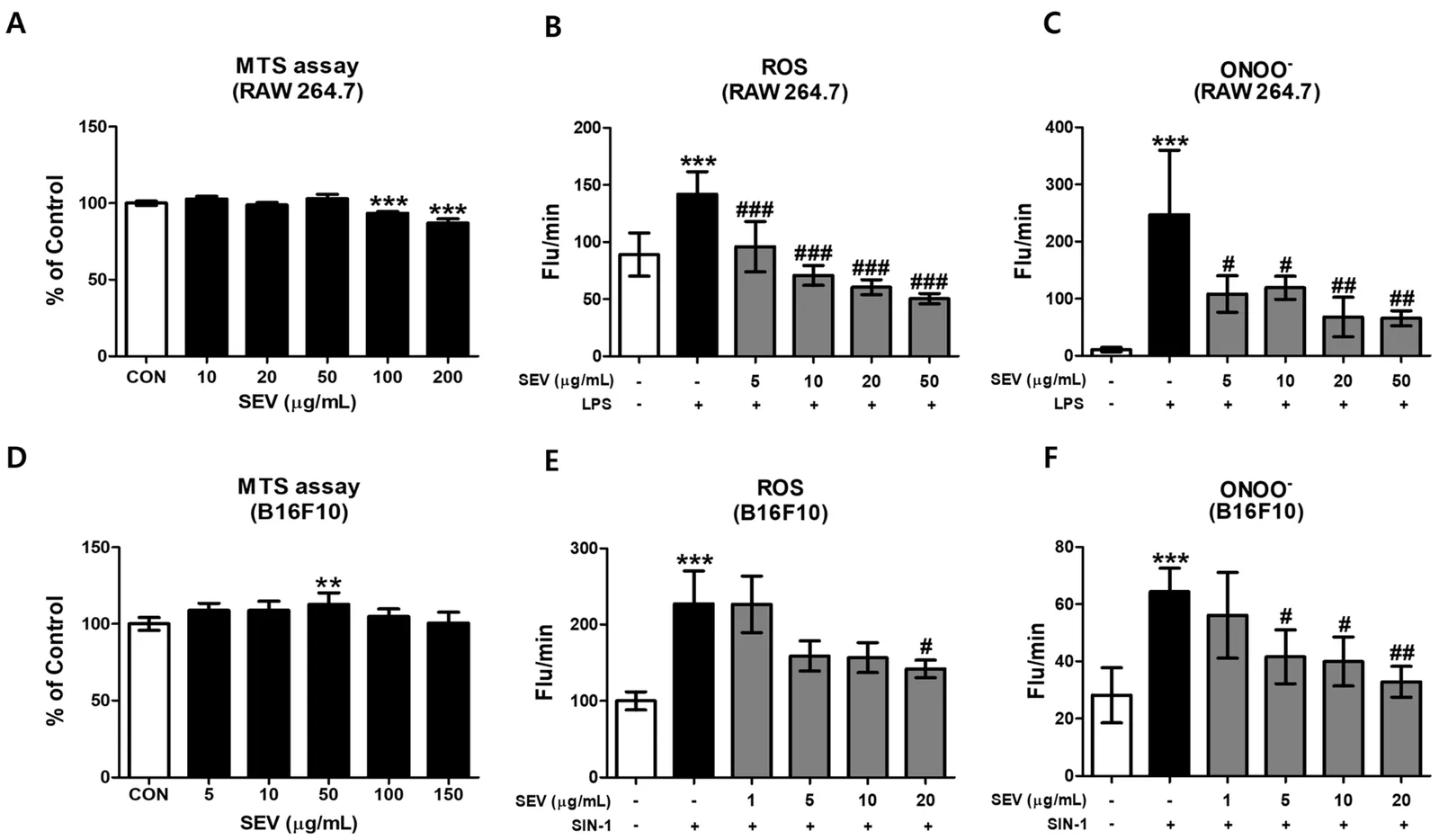
Effects of SEVs on cell viability, antioxidant activity, and inflammation. (**A**) RAW 264.7 cells were treated with various concentrations of SEVs (10–200 μg/mL) for 24 h, and cell viability was measured. (**B**,**C**) To evaluate the antioxidant effects of SEVs, RAW 264.7 cells were treated with various concentrations of SEVs (5–50 μg/mL) and subsequently stimulated with LPS. (**D**) B16F10 cells were treated with various concentrations of SEVs (5–150 μg/mL) for 24 h, and cell viability was measured. (**B**,**E**) ROS and (**C**,**F**) ONOO^−^ production were measured in LPS-stimulated RAW 264.7 cells and SIN-1-stimulated B16F10 cells using DCFDA and DHR123, respectively. “+” and “−” indicate the presence and absence of the indicated treatment, respectively. Data are expressed as the mean ± standard deviation (SD) (*n* = 6). ** p < 0.01, *** p < 0.001 compared with the untreated control group, *^#^* *p* < 0.05, *^##^ p* < 0.01, *^###^ p* < 0.001 compared with the LPS- and SIN-1-treated groups.

**Figure 5:**
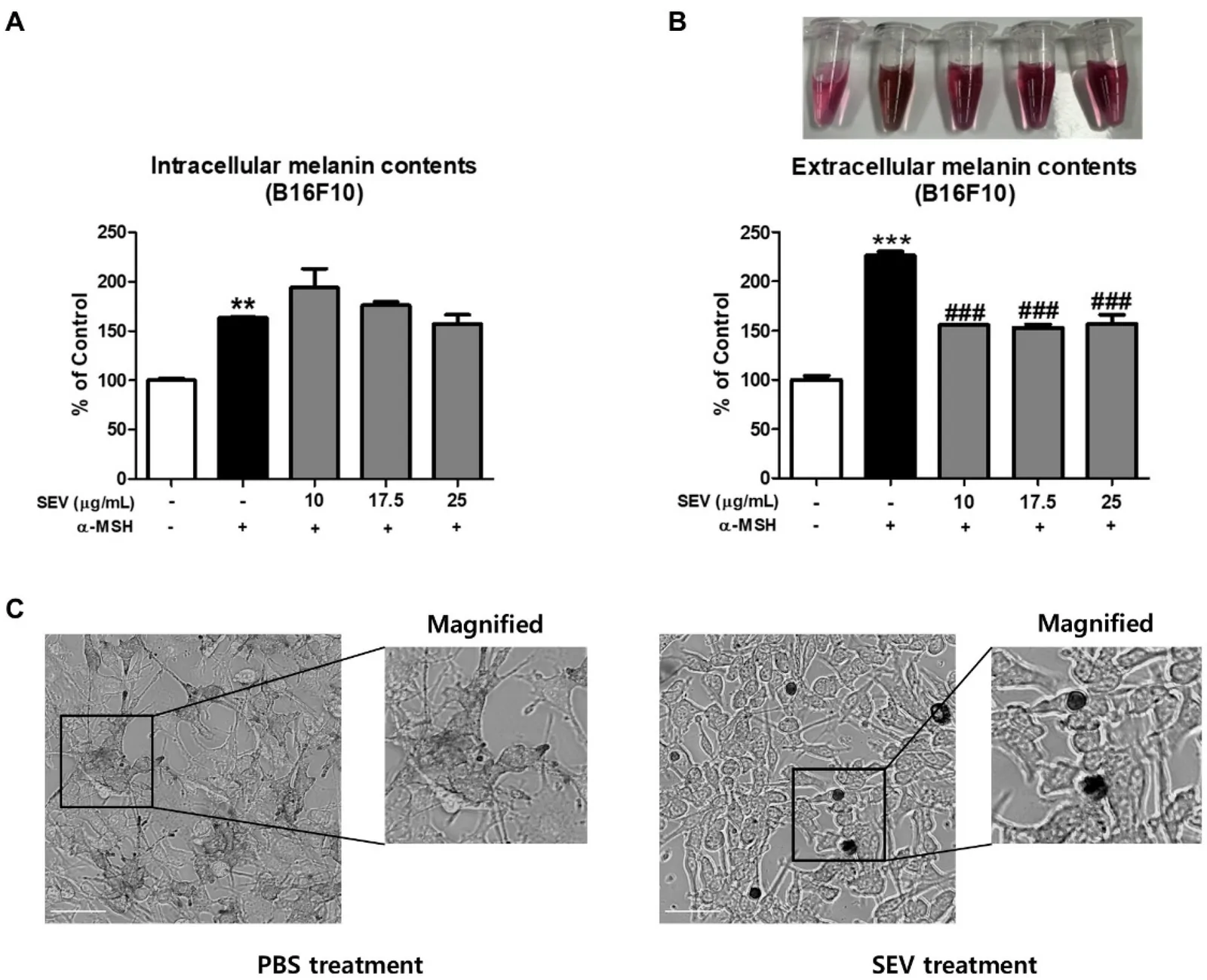
Effect of SEVs on intracellular and extracellular melanin content in B16F10 cells. (**A**,**B**) Intracellular and extracellular melanin content were measured in B16F10 cells pretreated with various concentrations of SEVs (10–25 µg/mL) for 1 h, followed by stimulation with α-MSH. (**C**) Microscopic observations of B16F10 cells treated with PBS and SEVs (17.5 µg/mL). Scale bar = 100 µm. ** *p* < 0.01, *** *p* < 0.001 compared with the untreated control group, ^###^ *p* < 0.001 compared with the α-MSH treatment group.

**Figure 6:**
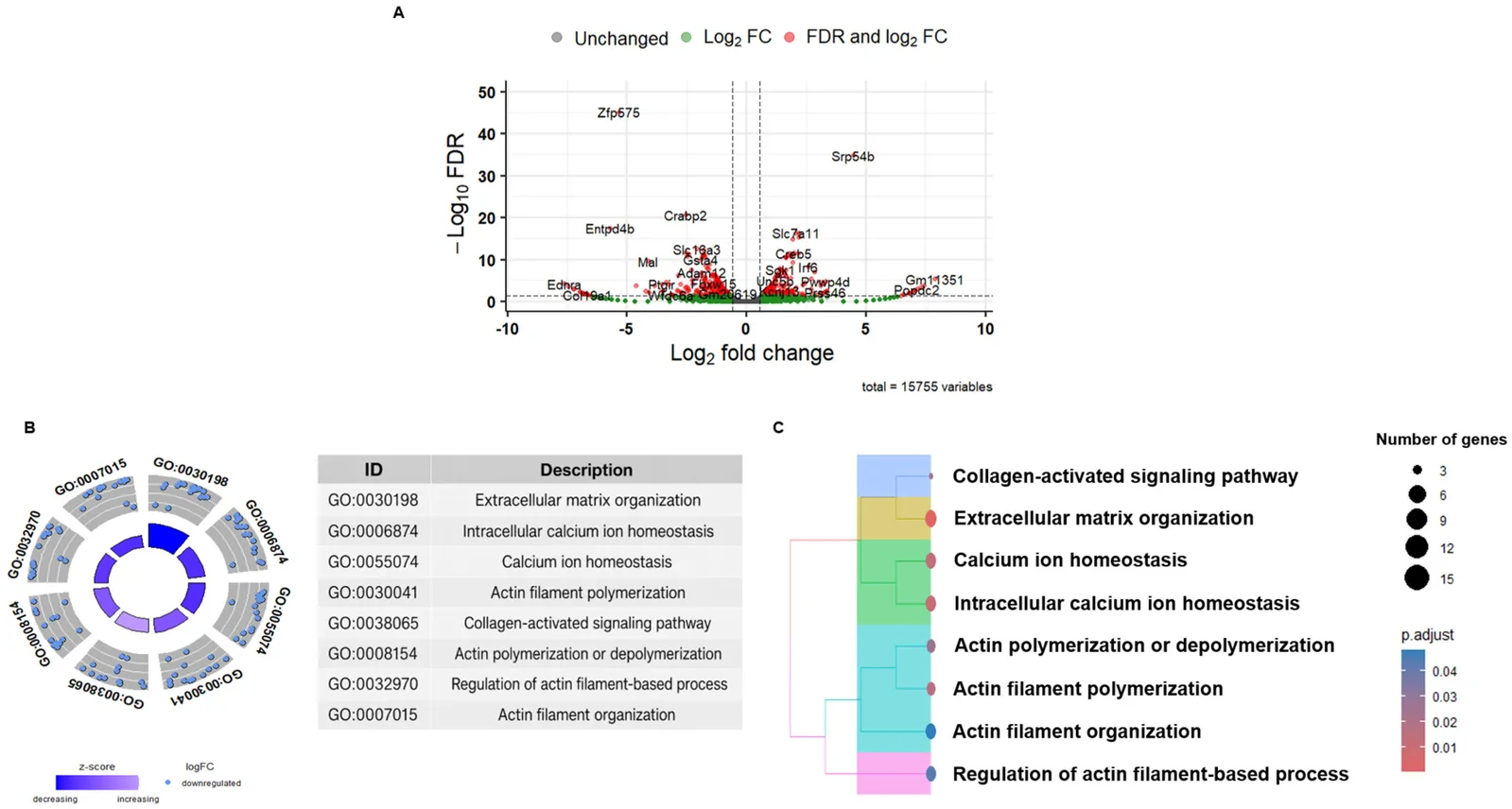
Differentially expressed genes and GO analysis in response to SEVs treatment in α-MSH-stimulated B16F10 cells. (**A**) Volcano plot of DEG in α-MSH stimulated B16F10 cells treated with SEVs. (**B**) GO circle plot of enriched GO terms. (**C**) Treeplots of enriched GO terms. Differentially expressed genes were identified using thresholds of |log_2_FC| ≥ 0.58 and FDR < 0.05.

**Figure 7:**
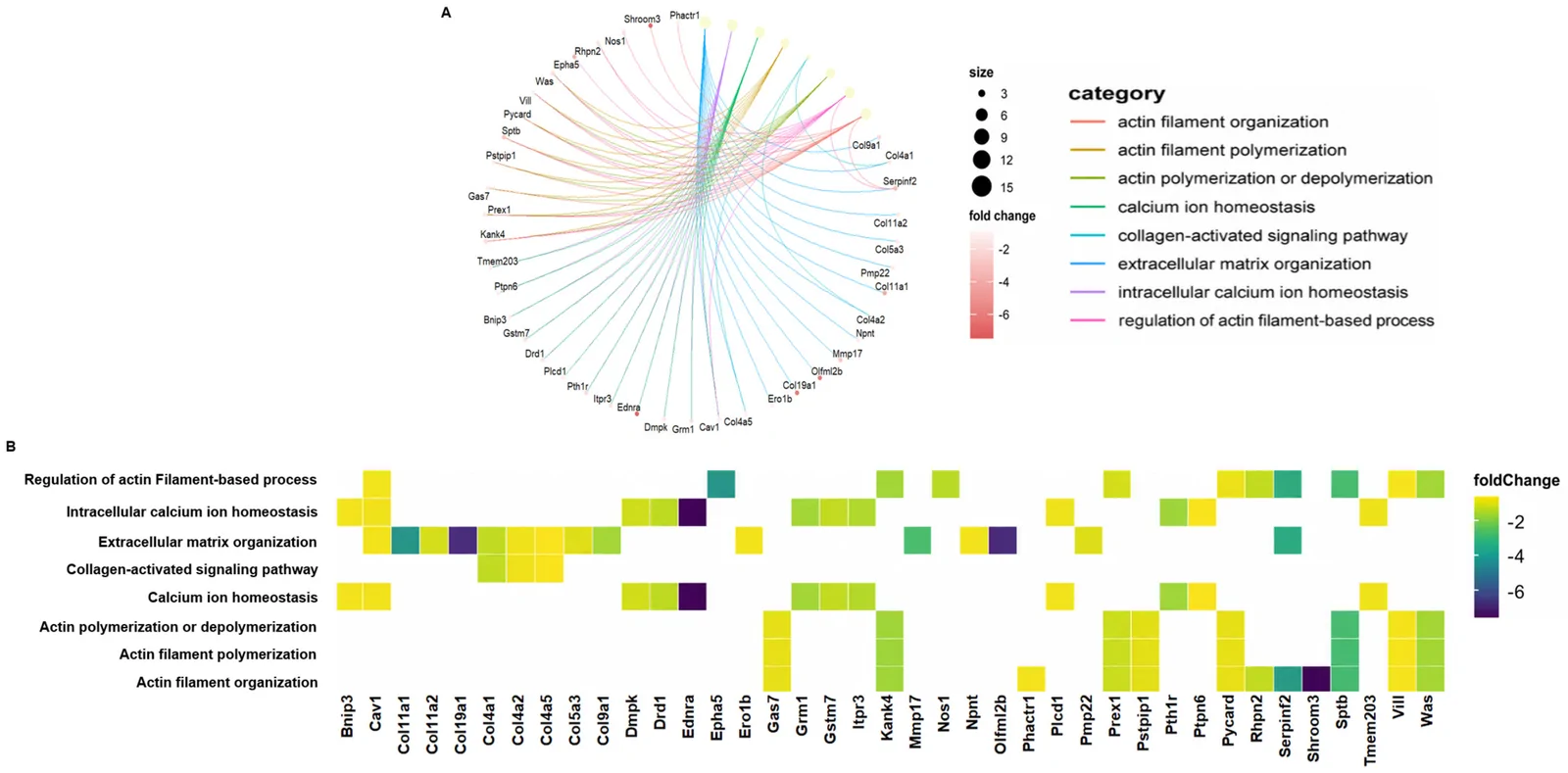
Network diagram and heatmap analysis of factors associated with GO terms in α-MSH-stimulated B16F10 cells treated with SEVs. (**A**) Network diagram illustrating relationships among GO terms. (**B**) Heatmap displaying gene expression patterns of factors associated with the identified GO terms. The analysis was performed using thresholds of |log_2_FC| ≥ 0.58 and FDR < 0.05.

**Figure 8:**
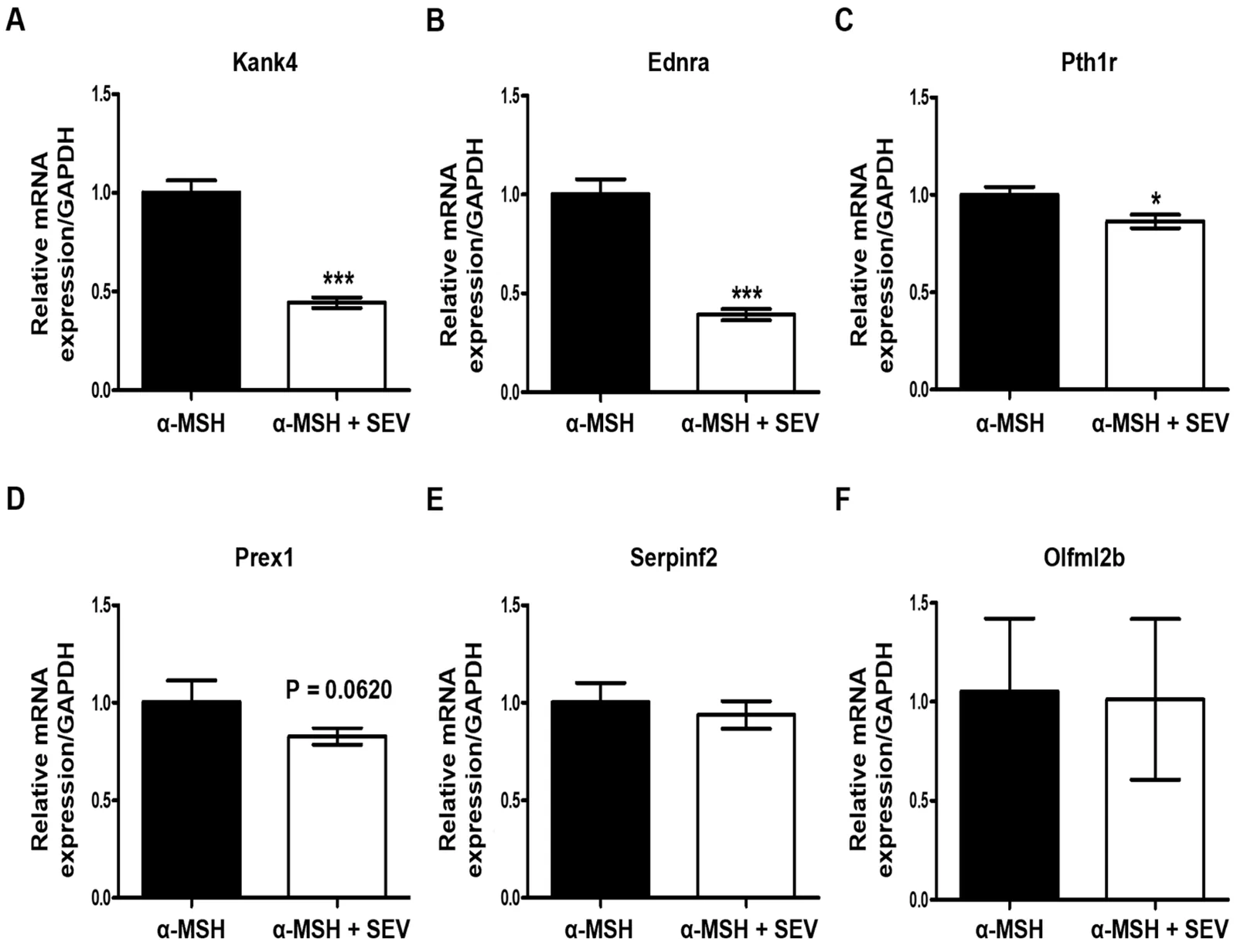
RT-qPCR assessment of genes related to GO analysis by RT-qPCR. The mRNA expression levels of the six selected genes (**A**) Kank4, (**B**) Ednra, (**C**) Pth1r, (**D**) Prex1, (**E**) Serpinf2, and (**F**) Olfml2 b were confirmed by RT-qPCR. Data are expressed as mean ± standard deviation (SD) (*n* = 3). * *p* < 0.05, *** *p* < 0.001 compared with the α-MSH treatment group.

**Figure 9:**
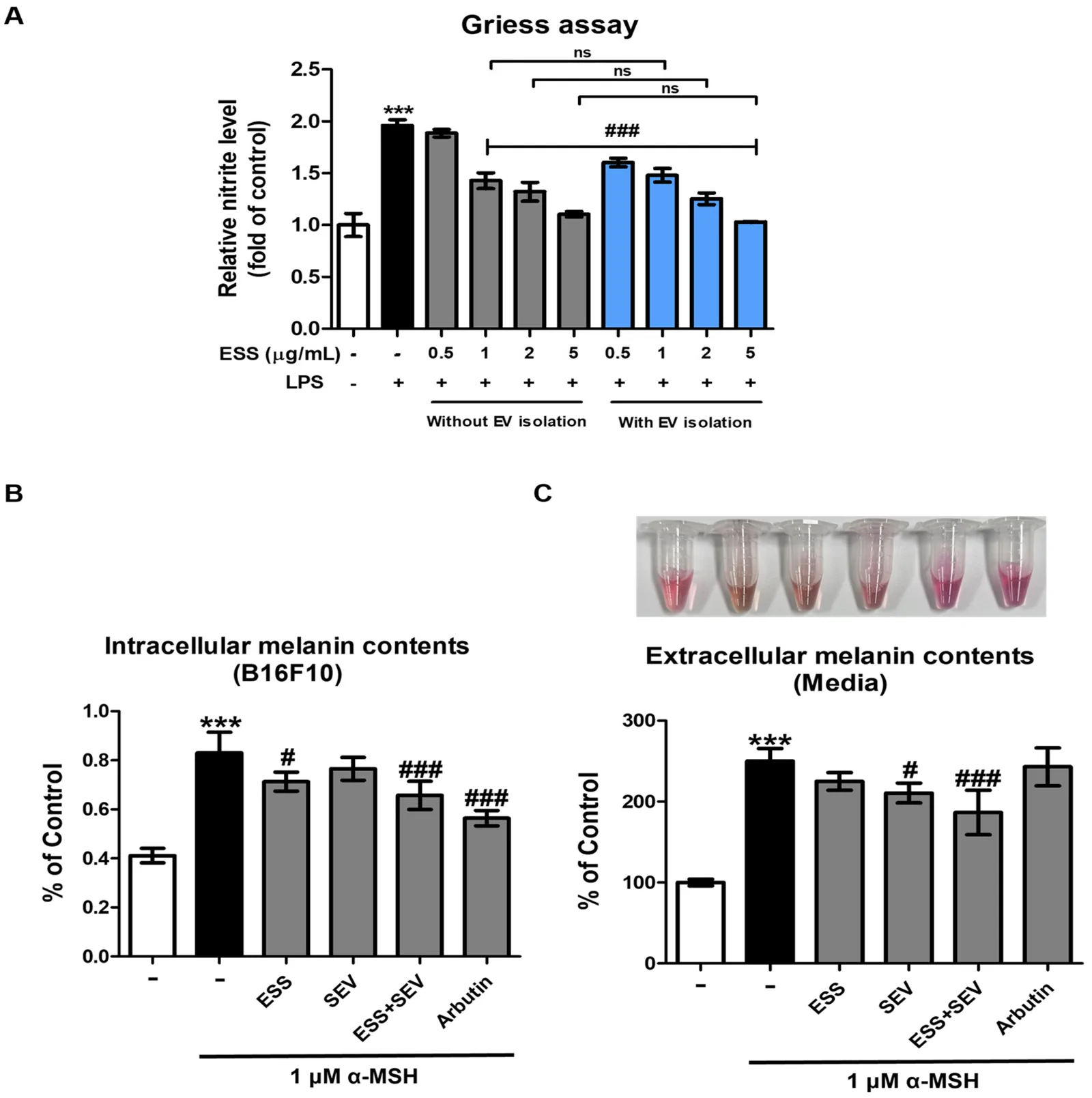
Comparison of the activities of ESS prepared with or without prior SEVs isolation and the complementary effects of SEVs and ESS on melanin regulation (**A**) Comparison of LPS-induced nitric oxide production using the Griess assay. RAW 264.7 macrophages were treated with ESS prepared from *S. serratifolium* with or without prior isolation of SEVs and subsequently stimulated with 500 ng/mL LPS. Nitrite accumulation in the culture medium was measured using the Griess reagent. Values were normalized to the untreated control group, which was set to 1. (**B**,**C**) Intracellular and extracellular melanin content were assessed in B16F10 cells treated with ESS (10 μg/mL), SEVs (17.5 µg/mL), and arbutin (500 µM) and then stimulated with α-MSH. Data are expressed as mean ± standard deviation (SD) (*n* = 6). Significant differences were analyzed using one-way ANOVA followed by the Tukey test. *** *p*< 0.001 compared with the untreated control group, ^#^ *p* < 0.05, ^###^ *p* < 0.001 compared with LPS-treated group and the α-MSH treatment group.

**Table 1:** Metabolites Identified in SEVs by Untargeted LC-MS/MS Analysis. The table summarizes the compound name, molecular formula, observed mass and *m*/*z*, retention time (RT), signal response, ionization mode (positive or negative), and detected adduct types. Compound identification was performed using the UNIFI software with a custom local library containing 816 phenolic compounds.

No.	Component Name	Formula	Observed Mass (Da)	Observed *m*/*z*	Observed RT (min)	Response	Ion Mode	Adducts
1	(-)-Epigallocatechin 3-O-glucuronide	C_21_H_22_O_13_	482.10564	483.11292	21.02150	4033.97583	POS	+H
2	Catechol	C_6_H_6_O_2_	110.03645	111.04373	17.92376	2247.89282	POS	+H
3	Dimethylmatairesinol	C_22_H_26_O_6_	386.17280	431.17101	15.06093	11,080.54980	NEG	+HCOO
			386.17280	387.18008	15.05672	54,006.15234	POS	+H
4	Peonidin 3-O-rutinoside	C_28_H_33_O_15_	609.17976	610.18704	22.59900	2056.28027	POS	+H
5	Sargachromanol C	C_27_H_40_O_3_	412.29702	457.29522	17.76102	26,345.08984	NEG	+HCOO
6	Sargachromanol D	C_27_H_40_O_4_	428.29201	473.29021	17.68658	52,349.30469	NEG	+HCOO
7	Sargachromanol H	C_27_H_40_O_4_	428.29214	473.29035	17.81546	39,943.79688	NEG	+HCOO
8	Sargachromanol J	C_27_H_42_O_4_	430.30740	475.30560	16.17831	2358.54932	NEG	+HCOO
9	Sargachromanol L	C_27_H_40_O_3_	412.29696	457.29516	18.01994	40,612.77344	NEG	+HCOO
10	Sargachromanol N	C_27_H_38_O_3_	410.28191	455.28011	18.63536	3539.70923	NEG	+HCOO
11	Sargachromenol	C_27_H_36_O_4_	424.26070	423.25343	18.86851	5900.15674	NEG	-H
			424.26137	425.26864	17.31724	7916.62646	POS	+H
12	Sargahydroquinoic Acid	C_27_H_38_O_4_	426.27584	471.27405	17.92998	2758.85693	NEG	+HCOO/-H
13	Vanillic acid 4-sulfate	C_8_H_8_O_7_S	247.99896	246.99169	2.72020	30,870.03320	NEG	-H

**Table 2:** Comparison of ESS yields from *S. serratifolium* based on the presence or absence of EV isolation.

EVs Isolation	Solvent of Extracts	*S. serratifolium* (g)	ESS (g)	Yields (%)
+	70% EtOH	12.16	1.98	16.3
−	70% EtOH	8.43	1.12	13.3

## Data Availability

The raw RNA-seq data generated in this study have been deposited in the NCBI Sequence Read Archive (SRA) under BioProject accession number PRJNA1515485 and are publicly available under SRA Run accession numbers SRR40287618 and SRR40287619.

## Supplementary Materials

The following supporting information can be downloaded at https://www.mdpi.com/article/10.3390/md24100344/s1, Table S1: LC gradient program used for chromatographic separation of SEV-derived metabolites; Figure S1: Effect of prolonged SEV treatment on B16F10 cell viability; Table S2: Primer sequences used for RT-qPCR validation of the RNA-seq data.

## References

[1] Raposo G., Stoorvogel W. (2013). Extracellular vesicles: Exosomes, microvesicles, and friends. J. Cell Biol..

[2] Abels E.R., Breakefield X.O. (2016). Introduction to Extracellular Vesicles: Biogenesis, RNA Cargo Selection, Content, Release, and Uptake. Cell Mol. Neurobiol..

[3] Meldolesi J. (2018). Exosomes and Ectosomes in Intercellular Communication. Curr. Biol..

[4] Zhang M., Viennois E., Xu C., Merlin D. (2016). Plant derived edible nanoparticles as a new therapeutic approach against diseases. Tissue Barriers.

[5] Lee R., Ko H.J., Kim K., Sohn Y., Min S.Y., Kim J.A., Na D., Yeon J.H. (2020). Anti-melanogenic effects of extracellular vesicles derived from plant leaves and stems in mouse melanoma cells and human healthy skin. J. Extracell. Vesicles.

[6] You J.Y., Kang S.J., Rhee W.J. (2021). Isolation of cabbage exosome-like nanovesicles and investigation of their biological activities in human cells. Bioact. Mater..

[7] Chen Q., Li Q., Liang Y., Zu M., Chen N., Canup B.S.B., Luo L., Wang C., Zeng L., Xiao B. (2022). Natural exosome-like nanovesicles from edible tea flowers suppress metastatic breast cancer via ROS generation and microbiota modulation. Acta Pharm. Sin. B.

[8] Hwang J.-H., Park Y.-S., Kim H.-S., Kim D.-H., Lee S.-H., Lee C.-H., Lee S.-H., Kim J.-E., Lee S., Kim H.M. (2023). Yam-derived exosome-like nanovesicles stimulate osteoblast formation and prevent osteoporosis in mice. J. Control. Release.

[9] Lian M.Q., Chng W.H., Liang J., Yeo H.Q., Lee C.K., Belaid M., Tollemeto M., Wacker M.G., Czarny B., Pastorin G. (2022). Plant-derived extracellular vesicles: Recent advancements and current challenges on their use for biomedical applications. J. Extracell. Vesicles.

[10] Jo S.-H., Kim S.-H., Kim C., Park S.-H. (2021). Characterization of Marine Organism Extracellular Matrix-Anchored Extracellular Vesicles and Their Biological Effect on the Alleviation of Pro-Inflammatory Cytokines. Mar. Drugs.

[11] Picciotto S., Barone M.E., Fierli D., Aranyos A., Adamo G., Božič D., Romancino D.P., Stanly C., Parkes R., Morsbach S. (2021). Isolation of extracellular vesicles from microalgae: Towards the production of sustainable and natural nanocarriers of bioactive compounds. Biomater. Sci..

[12] Drori E., Patel D., Coopersmith S., Rahamim V., Drori C., Jadhav S.S., Avital R., Anker Y., Azagury A. (2024). Algae-based nanoparticles for oral drug delivery systems. Mar. Drugs.

[13] Garaeva L., Tolstyko E., Putevich E., Kil Y., Spitsyna A., Emelianova S., Solianik A., Yastremsky E., Garmay Y., Komarova E. (2025). Microalgae-derived vesicles: Natural nanocarriers of exogenous and endogenous proteins. Plants.

[14] Jang B., Chung H., Jung H., Song H.K., Park E., Choi H.S., Jung K., Choe H., Yang S., Oh E.S. (2021). Extracellular Vesicles from Korean Codium fragile and Sargassum fusiforme Negatively Regulate Melanin Synthesis. Mol. Cells.

[15] Batsukh S., Oh S., Lee J.M., Joo J.H.J., Son K.H., Byun K. (2024). Extracellular Vesicles from Ecklonia cava and Phlorotannin Promote Rejuvenation in Aged Skin. Mar. Drugs.

[16] Mattio L., Payri C.E. (2011). 190 Years of Sargassum Taxonomy, Facing the Advent of DNA Phylogenies. Bot. Rev..

[17] Saraswati, Giriwono P.E., Iskandriati D., Tan C.P., Andarwulan N. (2019). Sargassum Seaweed as a Source of Anti-Inflammatory Substances and the Potential Insight of the Tropical Species: A Review. Mar. Drugs.

[18] Kim H., Shin H.Y., Jeong E.-J., Lee H.-D., Hwang K.C., Yu K.-W., Lee S., Lee S. (2022). Antioxidant and Anti-Inflammatory Activities of Sargassum macrocarpum Extracts. Antioxidants.

[19] González-Garrido J.A., Gómez-García J.A., Hernández-Abreu O.I., Olivares-Corichi I.M., Pereyra-Vergara F., García-Sánchez J.R. (2024). Anticancer Activity of Sargassum fluitans Extracts in Different Cancer Cells. Anticancer Agents Med. Chem..

[20] Liu L., Heinrich M., Myers S., Dworjanyn S.A. (2012). Towards a better understanding of medicinal uses of the brown seaweed Sargassum in Traditional Chinese Medicine: A phytochemical and pharmacological review. J. Ethnopharmacol..

[21] Fitton J.H. (2003). Brown Marine Algae: A Survey of Therapeutic Potentials. Altern. Complement. Ther..

[22] Chávez V., Uribe-Martínez A., Cuevas E., Rodríguez-Martínez R.E., van Tussenbroek B.I., Francisco V., Estévez M., Celis L.B., Monroy-Velázquez L.V., Leal-Bautista R. (2020). Massive Influx of Pelagic Sargassum spp. on the Coasts of the Mexican Caribbean 2014–2020: Challenges and Opportunities. Water.

[23] Fidai Y., Dash J., Tompkins E., Tonon T. (2020). A systematic review of floating and beach landing records of Sargassum beyond the Sargasso Sea. Environ. Res. Commun..

[24] Ruiz-Ramírez J.D., Euán-Ávila J.I., Rivera-Monroy V.H. (2019). Vulnerability of Coastal Resort Cities to Mean Sea Level Rise in the Mexican Caribbean. Coast. Manag..

[25] Franks J., Johnson D., Ko D., Sanchez-Rubio G., Hendon J., Lay M. (2011). Unprecedented influx of pelagic Sargassum along Caribbean island coastlines during summer 2011. Proceedings of the 64th Gulf and Caribbean Fisheries Institute.

[26] Resiere D., Valentino R., Nevière R., Banydeen R., Gueye P., Florentin J., Cabié A., Lebrun T., Mégarbane B., Guerrier G. (2018). *Sargassum* seaweed on Caribbean islands: An international public health concern. Lancet.

[27] Lim S., Choi A.-H., Kwon M., Joung E.-J., Shin T., Lee S.-G., Kim N.-G., Kim H.-R. (2019). Evaluation of antioxidant activities of various solvent extract from *Sargassum serratifolium* and its major antioxidant components. Food Chem..

[28] Kwon M., Lim S.-J., Joung E.-J., Lee B., Oh C.-W., Kim H.-R. (2018). Meroterpenoid-rich fraction of an ethanolic extract from *Sargassum serratifolium* alleviates obesity and non-alcoholic fatty liver disease in high fat-fed C57BL/6J mice. J. Funct. Foods.

[29] Kim M.J., Ryu H., Jeong H.H., Van J.Y., Hwang J.Y., Kim A.R., Seo J., Moon K.M., Jung W.K., Lee B. (2024). The beneficial effects of ethanolic extract of *Sargassum serratifolium* in DNCB-induced mouse model of atopic dermatitis. Sci. Rep..

[30] Ryu H., Jeong H.H., Kim M.-J., Lee S., Jung W.-K., Lee B. (2024). Modulation of macrophage transcript and secretion profiles by *Sargassum serratifolium* extract is associated with the suppression of muscle atrophy. Sci. Rep..

[31] Azam M.S., Kwon M., Choi J., Kim H.-R. (2018). Sargaquinoic acid ameliorates hyperpigmentation through cAMP and ERK-mediated downregulation of MITF in α-MSH-stimulated B16F10 cells. Biomed. Pharmacother..

[32] Meredith P., Sarna T. (2006). The physical and chemical properties of eumelanin. Pigment Cell Res..

[33] Brenner M., Hearing V.J. (2008). The protective role of melanin against UV damage in human skin. Photochem. Photobiol..

[34] Bastiaens M., Ter Huurne J., Gruis N., Bergman W., Westendorp R., Vermeer B.J., Bouwes Bavinck J.N. (2001). The melanocortin-1-receptor gene is the major freckle gene. Hum. Mol. Genet..

[35] Iriyama S., Ono T., Aoki H., Amano S. (2011). Hyperpigmentation in human solar lentigo is promoted by heparanase-induced loss of heparan sulfate chains at the dermal-epidermal junction. J. Dermatol. Sci..

[36] Dabas G., Vinay K., Parsad D., Kumar A., Kumaran M.S. (2020). Psychological disturbances in patients with pigmentary disorders: A cross-sectional study. J. Eur. Acad. Dermatol. Venereol..

[37] D’Mello S.A.N., Finlay G.J., Baguley B.C., Askarian-Amiri M.E. (2016). Signaling Pathways in Melanogenesis. Int. J. Mol. Sci..

[38] Ito S., Wakamatsu K. (2008). Chemistry of mixed melanogenesis--pivotal roles of dopaquinone. Photochem. Photobiol..

[39] Roh E., Yun C.-Y., Young Yun J., Park D., Doo Kim N., Yeon Hwang B., Jung S.-H., Park S.K., Kim Y.-B., Han S.-B. (2013). CAMP-Binding Site of PKA as a Molecular Target of Bisabolangelone against Melanocyte-Specific Hyperpigmented Disorder. J. Investig. Dermatol..

[40] Seo J., Hwang Y.-H., Lee A., Moon K.M., Van J.Y., Jeong H.H., Ryu H., Xi H.P., Lee B. (2024). Ethanolic Extract of Bergenia purpurascens Exhibits Antimelanogenic Effects in B16F10 Cells through Multiple Mechanisms That Suppress Tyrosinase. J. Agric. Food Chem..

[41] Bento-Lopes L., Cabaço L.C., Charneca J., Neto M.V., Seabra M.C., Barral D.C. (2023). Melanin’s Journey from Melanocytes to Keratinocytes: Uncovering the Molecular Mechanisms of Melanin Transfer and Processing. Int. J. Mol. Sci..

[42] Fukuda M., Kuroda T.S., Mikoshiba K. (2002). Slac2-a/Melanophilin, the Missing Link between Rab27 and Myosin Va: IMPLICATIONS OF A TRIPARTITE PROTEIN COMPLEX FOR MELANOSOME TRANSPORT. J. Biol. Chem..

[43] Seiberg M., Paine C., Sharlow E., Andrade-Gordon P., Costanzo M., Eisinger M., Shapiro S.S. (2000). Inhibition of melanosome transfer results in skin lightening. J. Invest. Dermatol..

[44] Moreiras H., Pereira F.J.C., Neto M.V., Bento-Lopes L., Festas T.C., Seabra M.C., Barral D.C. (2020). The exocyst is required for melanin exocytosis from melanocytes and transfer to keratinocytes. Pigment Cell Melanoma Res..

[45] Azam M.S., Kim J.I., Choi C.G., Choi J., Lee B., Kim H.R. (2021). Sargahydroquinoic Acid Suppresses Hyperpigmentation by cAMP and ERK1/2-Mediated Downregulation of MITF in α-MSH-Stimulated B16F10 Cells. Foods.

[46] Wu X., Bowers B., Rao K., Wei Q., Hammer J.A. (1998). Visualization of melanosome dynamics within wild-type and dilute melanocytes suggests a paradigm for myosin V function In vivo. J. Cell Biol..

[47] Jiang M., Paniagua A.E., Volland S., Wang H., Balaji A., Li D.G., Lopes V.S., Burgess B.L., Williams D.S. (2020). Microtubule motor transport in the delivery of melanosomes to the actin-rich apical domain of the retinal pigment epithelium. J. Cell Sci..

[48] Bahadoran P., Aberdam E., Mantoux F., Buscà R., Bille K., Yalman N., de Saint-Basile G., Casaroli-Marano R., Ortonne J.P., Ballotti R. (2001). Rab27a: A key to melanosome transport in human melanocytes. J. Cell Biol..

[49] Tienda-Vázquez M.A., Hanel J.M., Márquez-Arteaga E.M., Salgado-Álvarez A.P., Scheckhuber C.Q., Alanis-Gómez J.R., Espinoza-Silva J.I., Ramos-Kuri M., Hernández-Rosas F., Melchor-Martínez E.M. (2023). Exosomes: A Promising Strategy for Repair, Regeneration and Treatment of Skin Disorders. Cells.

[50] Seong Choi K., Shin T.S., Chun J., Ahn G., Jeong Han E., Kim M.J., Kim J.B., Kim S.H., Kho K.H., Heon Kim D. (2022). Sargahydroquinoic acid isolated from *Sargassum serratifolium* as inhibitor of cellular basophils activation and passive cutaneous anaphylaxis in mice. Int. Immunopharmacol..

[51] Zhang H., Chen Z., Zhang A., Gupte A.A., Hamilton D.J. (2022). The Role of Calcium Signaling in Melanoma. Int. J. Mol. Sci..

[52] Devi S., Kedlaya R., Maddodi N., Bhat K.M., Weber C.S., Valdivia H., Setaluri V. (2009). Calcium homeostasis in human melanocytes: Role of transient receptor potential melastatin 1 (TRPM1) and its regulation by ultraviolet light. Am. J. Physiol. Cell Physiol..

[53] Manning D., Dart C., Evans R.L. (2022). Store-operated calcium channels in skin. Front. Physiol..

[54] Mohan S., Nair A., Poornima M.S., Raghu K.G. (2023). Vanillic acid mitigates hyperinsulinemia induced ER stress mediated altered calcium homeostasis, MAMs distortion and surplus lipogenesis in HepG2 cells. Chem. Biol. Interact..

[55] Rinaldi D.E., Ontiveros M.Q., Saffioti N.A., Vigil M.A., Mangialavori I.C., Rossi R.C., Rossi J.P., Espelt M.V., Ferreira-Gomes M.S. (2021). Epigallocatechin 3-gallate inhibits the plasma membrane Ca^2+^-ATPase: Effects on calcium homeostasis. Heliyon.

[56] Gwon W.-G., Lee S.-G., Kim J.-I., Kim Y.-M., Kim S.-B., Kim H.-R. (2019). Hexane fraction from the ethanolic extract of *Sargassum serratifolium* suppresses cell adhesion molecules via regulation of NF-κB and Nrf2 pathway in human umbilical vein endothelial cells. Fish. Aquat. Sci..

[57] Lee M.-K., Ryu H., Jeong H.H., Lee B. (2023). Brassinin Abundant in Brassicaceae Suppresses Melanogenesis through Dual Mechanisms of Tyrosinase Inhibition. Foods.

[58] Moon K.M., Yang J.-H., Lee M.-K., Kwon E.-B., Baek J., Hwang T., Kim J.-I., Lee B. (2022). Maclurin Exhibits Antioxidant and Anti-Tyrosinase Activities, Suppressing Melanogenesis. Antioxidants.

[59] Moon K.M., Lee B., Kim D.H., Chung H.Y. (2020). FoxO6 inhibits melanogenesis partly by elevating intracellular antioxidant capacity. Redox Biol..

[60] Kim D., Paggi J.M., Park C., Bennett C., Salzberg S.L. (2019). Graph-based genome alignment and genotyping with HISAT2 and HISAT-genotype. Nat. Biotechnol..

[61] Liao Y., Smyth G.K., Shi W. (2014). FeatureCounts: An efficient general purpose program for assigning sequence reads to genomic features. Bioinformatics.

