# Supplementary material for: Inhibitory Effects of Extracellular Vesicles Derived from Sargassum serratifolium on Melanogenesis Partially by Suppressing Melanin Transport
Source: Mar Drugs. 2026 Sep 30;24(10):344. doi: 10.3390/md24100344 (PMC13706142; doi:10.3390/md24100344)
Supplement: Supplementary file 1 [file marinedrugs-24-00344-s001.zip › marinedrugs-4479124-supplementary.pdf]

Time (min)	A	B
0	98	2
2	98	2
12	65	35
20	5	95
22	5	95
22.01	98	2
25	98	2

Supplementary Table S1 LC gradient program used for chromatographic separation of SEV-derived metabolites. Mobile phase A consisted of water LC gradient program used for chromatographic separation of metabolites extracted from SEVs. Mobile phase A consisted of water with 0.1% formic acid, and mobile phase B consisted of acetonitrile with 0.1% formic acid. The gradient elution was carried out over 25 minutes with the following program:0–2 min, 2% B;2–12 min, linear increase to 35% B;12–20 min, linear increase to 95% B;20–22 min, hold at 95% B;22–22.01 min, decrease to 2% B;22.01–25 min, re-equilibration at 2% B. The flow rate was maintained at 0.3 mL/min throughout the run.

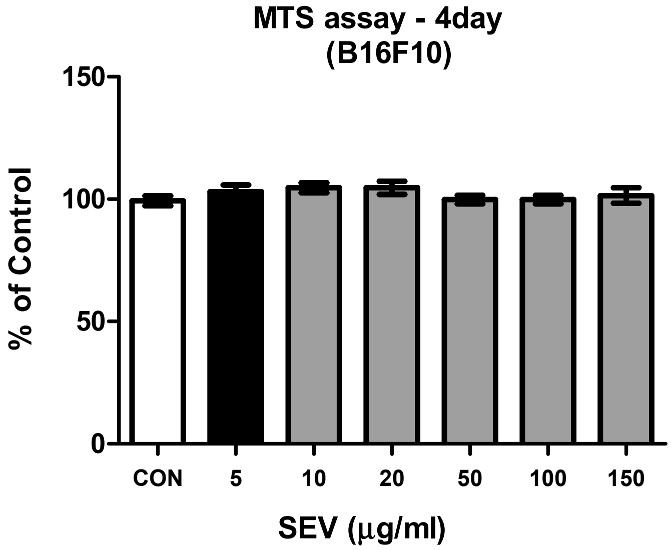

Supplementary Figure S1 **Effect of prolonged SEV treatment on B16F10 cell viability.** B16F10 cells were treated with the indicated concentrations of SEVs for 4 days, and cell viability was determined using the MTS assay. Data are presented as the mean ± SD from three independent experiments. No significant differences were observed compared with the untreated control group.

Gene	Forward primer	Reverse primer
Kank4	GTGTAGCAAGCAGGTAGGTC	GCCATTCTCTCTAGACATGC
**Ednra**	TCTTCTACACCCTCATGACC	GGCTTAAGTGAAGAGGGAAC
**Pth1r**	GTGCTCTACTCTGGCTTCAC	TAAGTACAGTCCCTCCACCA
**Prex1**	GAAGAACAAGGCTGTGGTAG	GAGCAGAAGAACTGGTTGAG
**Serpinf2**	AACCTGACCTGGGATACTCT	GCTCCATGGTAGACTGATGT
**Olfml2b**	CCCTCTCTACAATCACAGGA	GCTGTATGGAAGCTTGTAGG

Supplementary Table S2. Primer sequences used for the qRT-PCR validation of RNA-seq data. To validate the differential gene expression results obtained from the RNA sequencing (RNA-seq) analysis, six representative genes were selected for quantitative real-time PCR (qRT-PCR) verification. This table details the specific forward and reverse primer sequences used for the amplification of these six target genes.
